# Kaposi's Sarcoma Herpesvirus K15 Protein Contributes to Virus-Induced Angiogenesis by Recruiting PLCγ1 and Activating NFAT1-dependent RCAN1 Expression

**DOI:** 10.1371/journal.ppat.1002927

**Published:** 2012-09-27

**Authors:** Kiran Bala, Raffaella Bosco, Silvia Gramolelli, Darya A. Haas, Semra Kati, Marcel Pietrek, Anika Hävemeier, Yuri Yakushko, Vivek Vikram Singh, Oliver Dittrich-Breiholz, Michael Kracht, Thomas F. Schulz

**Affiliations:** 1 Institute of Virology, Hannover Medical School, Hannover, Germany; 2 Institute of Biochemistry, Hannover Medical School, Hannover, Germany; 3 Institute of Pharmacology, Justus-Liebig-Universität Giessen, Giessen, Germany; University of Washington, United States of America

## Abstract

Kaposi's Sarcoma (KS), caused by Kaposi's Sarcoma Herpesvirus (KSHV), is a highly vascularised angiogenic tumor of endothelial cells, characterized by latently KSHV-infected spindle cells and a pronounced inflammatory infiltrate. Several KSHV proteins, including LANA-1 (ORF73), vCyclin (ORF72), vGPCR (ORF74), vIL6 (ORF-K2), vCCL-1 (ORF-K6), vCCL-2 (ORF-K4) and K1 have been shown to exert effects that can lead to the proliferation and atypical differentiation of endothelial cells and/or the secretion of cytokines with angiogenic and inflammatory properties (VEGF, bFGF, IL6, IL8, GROα, and TNFβ). To investigate a role of the KSHV K15 protein in KSHV-mediated angiogenesis, we carried out a genome wide gene expression analysis on primary endothelial cells infected with KSHV wildtype (KSHVwt) and a KSHV K15 deletion mutant (KSHVΔK15). We found RCAN1/DSCR1 (Regulator of Calcineurin 1/Down Syndrome critical region 1), a cellular gene involved in angiogenesis, to be differentially expressed in KSHVwt- *vs* KSHVΔK15-infected cells. During physiological angiogenesis, expression of RCAN1 in endothelial cells is regulated by VEGF (vascular endothelial growth factor) through a pathway involving the activation of PLCγ1, Calcineurin and NFAT1. We found that K15 directly recruits PLCγ1, and thereby activates Calcineurin/NFAT1-dependent RCAN1 expression which results in the formation of angiogenic tubes. Primary endothelial cells infected with KSHVwt form angiogenic tubes upon activation of the lytic replication cycle. This effect is abrogated when K15 is deleted (KSHVΔK15) or silenced by an siRNA targeting the K15 expression. Our study establishes K15 as one of the KSHV proteins that contribute to KSHV-induced angiogenesis.

## Author Summary

Kaposi's Sarcoma Herpesvirus (KSHV) causes a multifocal angio-proliferative neoplasm, Kaposi's Sarcoma (KS), whose development involves angiogenic growth factors and cytokines. The K15 protein of KSHV upregulates the host factor RCAN1/DSCR1. RCAN1/DSCR1 has been implicated in angiogenesis but its role in KS has never been investigated. In this study we show that the increased expression of RCAN1/DSCR1 in KSHV-infected endothelial cells depends on K15 and that K15, by recruiting PLCγ1, activates PLCγ1, Calcineurin and NFAT1 to induce RCAN1/DSCR1 expression and capillary tube formation. Deleting the K15 gene from the viral genome, or silencing its expression with siRNA, reduces the ability of KSHV to induce angiogenesis in infected endothelial cells in tissue culture. These findings suggest that the K15 protein contributes to the angiogenic properties of this virus.

## Introduction

Kaposi's Sarcoma Herpesvirus (KSHV) or Human Herpesvirus 8 (HHV8) is a gammaherpesvirus first identified in Kaposi's Sarcoma (KS) biopsies [1]. Apart from being the etiological agent of the classic, AIDS-associated, endemic (African) and iatrogenic forms of Kaposi's sarcoma, it is also associated with two lymphoproliferative disorders, primary effusion lymphoma (PEL) [2] and multicentric Castleman's disease (MCD) [3]. KS is an angio-proliferative disease and the histology of this tumor is characterized by KSHV-infected spindle-shaped activated endothelial cells, vascular spaces and infiltrating inflammatory cells, in particular, monocytes and eosinophils. Atypical endothelial cells and infiltrating inflammatory cells predominate in the early stage of KS (“patch, plaque”), whereas the endothelial spindle cells, the hallmark of KS lesions, become more numerous in the later nodular stage (reviewed in [4], [5]). Angiogenic and inflammatory cytokines are thought to play an important role in KS pathogenesis [6]. In cell culture, KSHV-infected primary endothelial cells adopt a spindle like morphology, reminiscent of KS spindle cells seen in tumor biopsies [7]–[12]. Both *in vivo* and *in vitro*, the vast majority of endothelial spindle cells are latently infected with KSHV, but a small proportion of endothelial cells shows signs of productive viral replication [8], [9]. The formation of spindle cells is mainly due to the latent viral protein vFLIP [13], [14]. In addition, KSHV-infected primary vascular endothelial cells show evidence of differentiation into lymphatic endothelial cells, whereas lymphatic endothelial cells change their transcriptional program to resemble vascular endothelial cells [15]–[17]. Experimentally, KSHV has also been shown to induce neo-angiogenesis by inducing the formation of vascular tubes when KSHV-infected primary endothelial cells are plated on matrigel [18]. This phenomenon appears to be more pronounced after activation of the productive replication cycle [18]–[20]. When expressed in isolation, several viral proteins have been shown to play a role in the induction of angiogenesis, including latency-associated nuclear antigen 1 (LANA; open reading frame (ORF) 73), [21], [22], vGPCR, the viral homologue of a G-protein coupled receptor (ORF74) [23]–[29], viral interleukin 6 (vIL6; ORF-K2) [29]–[31], and two viral chemokine homologues (vCCL-1; ORF-K6, and vCCL-2; ORF-K4) [32]. In addition, the viral K1 protein has also been implicated in KSHV-mediated angiogenesis [19], [20], [33]. Furthermore, KSHV infection leads to the production of angiogenic and inflammatory cytokines like VEGF, bFGF, IL6, IL8, GRO-α and TNF-β/LTAα [31], [34]–[37]. Vascular endothelial growth factor (VEGF) is an important angiogenic growth factor that is expressed in KS lesions and can induce endothelial cell growth and angiogenesis. The biological effects of VEGF are mediated by cell surface receptors, VEGFR-1 (FLT1), VEGFR-2 (KDR), and VEGFR-3 (FLT4) [38]. Endothelial cells express both VEGFR-1 and VEGFR-2, with VEGFR-2 being the principal receptor through which VEGF signals are transmitted [39]. Binding of VEGF to VEGFR-2 results in the activation of intracellular signaling pathways including MAPKs and PI3K [38], [40]. In addition, VEGFR-2 also activates the Phospholipase C γ1 (PLCγ1) pathway leading to an increase in intracellular calcium and activation of protein kinase C (PKC), thereby inducing the expression of NFAT-dependent genes like DSCR1 (Down syndrome critical region 1, also called RCAN1 (Regulator of Calcineurin 1)) and Cox-2 (Cyclooxygenase 2) that have been shown to be involved in angiogenesis [41]–[45]. The human RCAN1/DSCR1 gene comprises 7 exons and exons 1–4 can be alternatively spliced to yield four transcripts (RCAN1.1 through RCAN1.4) [42]. Knockdown of RCAN1 has been shown to inhibit VEGF-mediated migration of endothelial cells *in vitro*, angiogenesis *in vivo* and thereby tumor growth [43], [44], [46].

In our previous studies we investigated the functional role of the KSHV K15 protein, a non-structural viral membrane protein. The ORF K15 is located between the terminal repeat region and ORF 75 at the ‘right’ end of the long unique coding region of the viral genome. ORF K15 consists of eight alternatively spliced exons. The main K15 protein is predicted to feature 12 transmembrane segments and a C-terminal cytoplasmic domain, which contains several putative signaling motifs such as two SH2-binding sites (Y^431^ASIL and Y^481^EEVL), a proline-rich SH3-binding site (P^387^PLP) and a tumor necrosis factor receptor-associated factor (TRAF)-binding site (A^473^TQPTDD) [47]–[49]. We have shown before that the K15 protein interacts with cellular proteins like TRAFs and members of the Src family of protein tyrosine kinases via its C-terminal domain [50], [51], thereby activating the MAP kinases c-jun-N-terminal kinase (JNK) 1 and extracellular signal-regulated kinase (ERK2), as well as the NFκB pathway resulting in the activation of AP-1 and NFAT-dependent gene expression [50], [52], [53]. When expressed in epithelial cells, K15 induces the production of several cytokines and chemokines, as well as cellular genes known to be involved in angiogenesis and cell invasion (e.g. RCAN1/DSCR1, MMP1, MMP2 and IL8) [52].

Here we investigated whether RCAN1/DSCR1 is regulated by K15 in the context of virus-infected endothelial cells and if K15 plays a role in KSHV-induced angiogenesis. Our results show that expression of RCAN1/DSCR1 is upregulated in KSHVwt-infected endothelial cells but not in cells infected with a K15 deletion mutant of KSHV. We further found that K15 directly interacts with PLCγ1 to activate the PLCγ1-Calcineurin-NFAT pathway and induces RCAN1/DSCR1 expression and thereby tubular morphogenesis in KSHV-infected human umbilical vein endothelial cells (HUVECs). Deletion of K15 from the viral genome, or silencing its expression with siRNA, reduces the ability of KSHV to induce angiogenesis in cultured endothelial cells. Our study establishes K15 as one of the KSHV proteins that contribute to KSHV-induced angiogenesis.

## Materials and Methods

### Cells and infection

HEK293 and HEK293T cells were cultured in Dulbecco's modified Eagle medium (DMEM) (Gibco) and Vero cells in Eagle minimal essential medium (MEM) (Biochrom), each supplemented with 10% FCS (Gibco), 50 IU/ml penicillin and 50 µg/ml streptomycin (Cytogen) in a 5% CO_2_ incubator. Human umbilical vein endothelial cells (HUVECs) were isolated from freshly obtained human umbilical cords by collagenase digestion of the interior of the umbilical vein as described previously [54] and were cultured in EGM2MV medium (Lonza) at 37°C in a 5% CO_2_ incubator. HEK293 and Vero clones, stably transfected (HEK293) or infected (Vero) with a bacterial artificial chromosome (BAC) carrying a wildtype or K15-deleted KSHV genome (see below), were cultured with additional 150 µg/ml hygromycin B (PAA). For infection with KSHV, HUVECs were cultured in medium without FCS. KSHV virus stocks, prepared as described below, were titrated on HEK293 cells. HUVECs were infected with a multiplicity of infection (m.o.i.) of 10 and were incubated on a shaker for 20 minutes followed by centrifugation for 30 minutes at 450×g. On the following day the medium was changed to medium containing FCS.

### Establishment of stable BACΔK15-containing HEK293 and Vero cell lines

A K15 deletion mutant (KSHVΔK15) was constructed in the KSHV bacterial artificial chromosome 36 (KSHVBAC36) [55] using ET recombination essentially as described in [14] by replacing nucleotide 135338 to 136900 of the KSHV genome with an rpsL/neomycin cassette. Details of the construction of KSHVΔK15 are available on request. To verify the integrity of the KSHVΔK15 genome, the entire BAC was sequenced on a Roche/454 next generation sequencer as previously described [56]. The sequence of KSHVΔK15 has been deposited in GenBank with the accession number JX228174.

4 µg of BAC DNA were used to transfect HEK293 cells at 60% confluence in a 6 well plate. For transfection, Lipofectamine 2000 (Invitrogen) was used according to the manufacturer's instructions. Transfected cells were monitored daily for the percentage of green fluorescent protein (GFP)-positive cells and two days after transfection cells were split 1∶2. One day later, 150 µg/mL hygromycin B was added to the medium, and the medium was changed every 2–3 days. Single clones were picked and propagated further under selection with hygromycin B. The clones were named 293-KSHVwt (for the wild type) and 293-KSHVΔK15 (for the deletion mutant of K15). For the establishment of KSHVΔK15-harboring Vero cells, cells in 48 well plates were infected with virus produced from HEK293 stable cell lines and kept under hygromycin B selection after reaching a confluence of 70–90%. Propagation of Vero cells for the establishment of stable cell lines was carried out as described above for HEK293 cells.

### Production of recombinant KSHV stocks

To produce virus stocks for infection of HUVECs, Vero cells containing KSHVwt or KSHVΔK15 genome in a BAC vector, or a recombinant KSHV (rKSHV.219) [57], were plated at 30 to 40% confluence in T-175 flasks. The following day, the medium was replaced with induction medium containing 1.25 mM sodium butyrate (Na-Bu) (Sigma) and 10% SF9 cell supernatant containing a baculovirus (a kind gift from J. Vieira) coding for KSHV RTA [52]. The supernatant was harvested 48 to 72 hours later and centrifuged at 5,000 g for 10 minutes and cell debris was cleared by filtration using 0.45 µm filters. For heat-inactivated virus stocks, the supernatant was heated at 65°C in a thermomixer for 1 hour and then centrifuged at 14,000 rpm for 10 minutes at 4°C to remove precipitates. The cleared supernatant was collected in centrifuge bottles (230 ml/bottle) and centrifuged at 15,000 rpm for 5 hours using a Type 19 rotor in a Beckman ultracentrifuge. The supernatant was then discarded and the pellet was resuspended in 300 µl medium overnight at 4°C. The resuspended virus was kept at 4°C.

For detection and quantification of the KSHV titers, 2×10^4^ HEK293 cells were plated per well of a 96-well plate and infected on the second day with serial dilutions of the centrifuged supernatants. GFP-positive cells were counted on day 2, and the infectious virus titer was calculated per ml.

### Retroviral vector construction, retrovirus production and transduction of HUVECs

The retroviral vector pSF91, a gift from Dr. C. Baum from the Department of Experimental Haematology, MHH, contains an internal ribosome entry site (IRES) upstream of the gene expressing enhanced GFP (eGFP). A K15 cDNA, codon-optimized for mammalian cells, was purchased from Gene Art Ltd, Regensburg (Germany) and cloned in the pSF91 vector using *Not1* sites to generate pSF91-K15-IRES-GFP.

One day prior to transfection, 4.2×10^6^ HEK293T cells were plated in a 10 cm dish. Transfection was carried out using a transfection medium containing HEPES (1 M) and chloroquine (25 µM). Subsequently, retroviral vector plasmids pSF91-IRES-GFP or pSF91-K15-IRES-GFP (5 µg) were co-transfected with packaging plasmids pM57DAW gag/pol (15 µg) and pRD114 vector (5 µg) expressing the envelope protein, using the calcium-phosphate transfection method. Twelve hours later the medium was replaced with fresh medium. Culture supernatant was harvested 36 and 48 hours post-transfection. Cell debris from the supernatant was cleared by filtration through 0.45 µm filters and then concentrated by ultracentrifugation at 10,000 rpm at 4°C for 16–18 hours using a SW 28 rotor in a Beckman ultracentrifuge. The pellet was resuspended in 200 µl of EGM2MV medium and aliquots were stored at −80°C. To infect HUVECs, 10 µl aliquots of pSF91-IRES-GFP and 50 µl aliquots of pSF91-K15-IRES-GFP virus stocks were inoculated on 1×10^5^ HUVECs in 6 well plates in the presence of polybrene (5 µg/ml). The medium was changed after 4 hours and 2 days after infection, the percentage of infected GFP-expressing HUVECs was estimated under a fluorescence microscope.

### Immunoblotting

Protein lysates from HUVECs were prepared in SDS sample buffer (62.5 mM Tris-HCl, pH 6.8, 2% (w/v) SDS, 10% glycerol, 50 mM DTT, 0.01% (w/v) bromophenol blue) containing β- mercaptoethanol for the detection of both PLCγ1 and phospho PLCγ1. For detection of all other proteins, lysates were prepared in RIPA100 buffer (20 mM Tris pH 7.5; 1 mM EDTA; 100 mM NaCl; 1% Triton-X100; 0.5% sodium deoxycholate, 0.1% SDS). For K15 proteins, lysates were not boiled prior to SDS-PAGE. Proteins were resolved by SDS-PAGE, and transferred onto nitrocellulose membranes (Amersham). Membranes were blocked with PBS-T 5% (w/v) milk. Proteins were detected using the following primary antibodies; a rabbit antibody to the cytoplasmic domain of K15 [48], [50], rabbit polyclonal to RCAN1/DSCR1 (D6694; Sigma), mouse monoclonal to NFAT1 (610702; BD Transductions Labs), rabbit polyclonal to Calcineurin pan A (#07-1491, Millipore), rabbit polyclonal to phospho-PLCγ1 (Tyr 783; #2821, Cell Signaling), rabbit polyclonal to total PLCγ1 (# 2822; Cell Signaling), rabbit polyclonal to VEGFR-1 (#2893, Cell Signaling), rabbit polyclonal to VEGFR-2 (#2479, Cell Signaling), rabbit polyclonal to VEGFR-3 (#2638, Cell Signaling) and mouse monoclonal to actin (Chemicon). Membranes were washed 3 times with PBS-T or TBS-T (for phospho-specific antibodies), and incubated with peroxidase-conjugated secondary antibodies. Proteins were detected using a standard enhanced chemiluminescence (ECL) detection kit (Thermo scientific).

### Microarray-based mRNA expression analysis

Total RNA was extracted with the RNeasy Micro Kit (Qiagen) according to the manufacturer's recommendation and was subjected to microarray analysis using “Whole Human Genome Microarray” (G4112F, AMADID 014850, Agilent Technologies). This microarray contains 45015 oligonucleotide probes covering roughly 31000 human transcripts. Synthesis of cRNA was performed with the “Quick Amp Labeling kit, one color” (Agilent Technologies) according to the manufacturer's recommendation. cRNA fragmentation, hybridization and washing steps were performed exactly as recommended: “One-Color Microarray-Based Gene Expression Analysis V5.7” (see http://www.agilent.com for details) except that 2.5 µg of each labeled cRNA sample were used for hybridization. Slides were scanned on the Agilent Micro Array Scanner G2505 B at two different PMT settings (100% and 5%) to increase the dynamic range of the measurements (extended dynamic range mode). Data extraction was performed with the “Feature Extraction Software V9.5.3.1” by using the recommended default extraction protocol file: GE1-v5_95_Feb07.xml.

**Figure 1:**
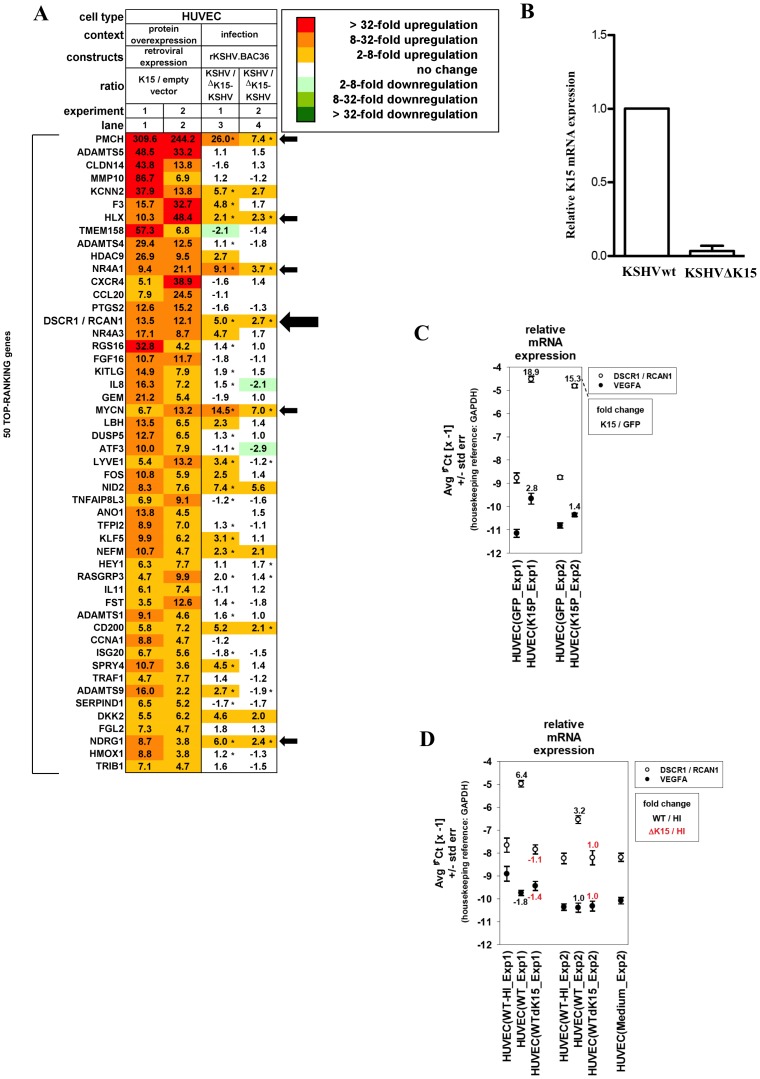
K15 dependent cellular gene expression in HUVECs. (A) Microarray analysis of cellular mRNAs upregulated in HUVECs by K15 overexpression or after KSHV infection. HUVECs were transduced with a K15-expression vector (lanes 1–2) or infected with either KSHVwt or KSHVΔK15 (lanes 3–4). Depicted are the top-ranking 50 transcripts most strongly upregulated by K15 overexpression, showing an at least two-fold induction by K15 overexpression in each of two experiments performed using cells from two healthy donors (lanes 1–2). Asterisks next to fold change values within heatmap lanes 3–4 indicate genes that were induced more than two-fold in KSHVwt-infected cells compared to cells mock infected with heat-inactivated virus. Empty cells in the heatmap correspond to undetectable mRNA levels in both of the two samples compared in each lane. Arrows indicate cellular genes, inducible upon KSHVwt infection with more than two-fold elevated expression in KSHVwt- relative to KSHVΔK15-infected cells in each of the two experiments performed. (B) Quantitative PCR analysis of K15 transcript in mRNA derived from KSHVwt- or KSHVΔK15-infected cells used for the microarray experiment. (C) Quantitative PCR analysis of RCAN1/DSCR1 and VEGFA transcripts in HUVECs transduced with a retroviral K15 expression vector or with an empty control vector. (D) HUVECs from two healthy donors (Exp1–2) were infected with heat-inactivated, KSHVwt, or KSHVΔK15 and were lysed 4 days after infection. RNA was extracted, reversely transcribed and subjected to quantitative TaqMan-based PCR analysis. mRNA fold change values of KSHVwt-infected versus mock-infected (with heat-inactivated KSHVwt = HI) cells were depicted in black, values of KSHVΔK15- infected versus mock-infected cells were depicted in red. Input mRNA samples are identical to samples used in microarray experiments (compare panel A lanes 3–4).

Processed intensity values of the green channel (“gProcessedSignal” or “gPS”) were normalized by global linear scaling: All gPS values of one sample were multiplied by an array-specific scaling factor. This scaling factor was calculated by dividing a “reference 75th Percentile value” (set as 1500 for the whole series) by the 75th Percentile value of the particular Microarray (“Array i” in the formula shown below). Accordingly, normalized gPS values for all samples (microarray data sets) were calculated by the following formula: *normalized gPS_Array i_ = gPS_Array i_×(1500/75^th^ Percentile_Array i_)*. A lower intensity threshold was defined as 1% of the reference 75th Percentile value ( = 15). All of those normalized gPS values that fell below this intensity border, were substituted by the respective surrogate value of 15. Calculation of ratio values of relative gene expression and data filtering were performed using excel macros or R-Scripts. Filters were set to exclude all technically impaired spots and poorly annotated, poorly characterized, or non-coding transcripts. For K15-overexpression studies, ratio values were calculated from processed signal intensities of “K15 overexpressing/empty vector transduced” HUVEC samples, for experiments in the context of KSHV infection, ratio values were calculated from “KSHVwt-infected/KSHVΔK15-infected” HUVEC samples. Relative fold differences in mRNA expression were color-coded appropriately as indicated in the legend of figure 1.

### TaqMan-based quantitative PCR analysis

The same total RNA samples as used in microarray experiments were reverse transcribed using 50 U of BioScript RNase H Low reverse transcriptase (BIO-27036, Bioline) in 20 µl reactions. The enzyme was finally inactivated for 10 minutes at 70°C. Aliquots of generated cDNA samples were used for real-time PCR with the ABI7500 FAST real-time PCR system (Applied Biosystems). Specific amplification was assured utilizing TaqMan probes and gene specific primers. Amplification was performed in 10 µl reactions with TaqMan Universal PCR Master Mix under recommended conditions (Applied Biosystems; #4364341). The following TaqMan gene expression assays (Applied Biosystems: #4331182) were used: Hs01120954_m1 (RCAN1); Hs00173626_m1 (VEGFA); Hs99999905_m1 (GAPDH). To detect K15 mRNA, primers 5′-CGGAAGAATCACGTGAAC-3′ (sense) and 5′-CGGTGTCTATACGGAAGG-3′ (antisense) and a dually labeled probe 5′-FAM-TCACCACAGCCAGACCAATCA-TAMRA-3′ were used. The average cycle-threshold value (Ct) for each individual amplification reaction was calculated from triplicate measurements by means of the instrument's software in “auto Ct” mode (7500 FAST System Software v.1.3.0).

### siRNA transfection of HUVECs

For most siRNA transfection experiments, HUVECs were transduced with pSF91-IRES-GFP (control vector) or pSF91-K15-IRES-GFP. Thirty hours after transduction, cells were transfected with 100 pmol siRNA using the Neon transfection system (Invitrogen) according to the manufacturer's instructions. The following siRNAs (siGENOME SMARTpool) were ordered from Dharmacon: Control siRNA (Non-Targeting siRNA pool #1, D-001206-13-05), siRCAN1 (M-004268-02), siNFATc2 (M-003603-02), siPLCG1 (M-003559-01), siRELA/p65 (M-003533-02), siCalcineurin (PPP3CA) (M-008300-02), siFLT1 (M-003136-03), siFLT4 (M-003138-02) and siKDR (M-003148-01). To selectively target RCAN1.1, siRNA was designed to target the exon 1 of RCAN1 (GCUUCAUUGACUGCGAGAUU). The RCAN1.4 isoform was specifically targeted by siRNA against exon 4 of RCAN1 (AGUGAUAUCUUCAGCGAAAUU). siRNA against K15 protein was targeted to exon 8 of K15 (CAACCACCUUGGCAAUAAU) and was also purchased from Dharmacon.

### *In-vitro* capillary tube formation assay

HUVECs were transduced with pSF91-K15-IRES-GFP or pSF91-IRES-GFP retrovirus stocks. Thirty hours later cells were washed twice with PBS and then incubated in basal medium (EBM2+2% FBS) overnight to starve the cells. Growth factor-reduced Matrigel (BD Biosciences) was added to the wells of a prechilled 96-well plate (65 µl/well). The plate was placed in a 37°C incubator for 30 min and the matrigel was allowed to solidify. To determine whether HUVECs, untransduced or transduced with retroviral vectors, could form angiogenic tubes in the absence or presence of VEGF, the cells were resuspended either in basal medium (EBM2+2% FBS) or basal medium containing VEGF-A (50 ng/ml). 1.6×10^4^ cells/ml (in 100 µl/well medium) were then plated on the solidified matrigel and incubated at 37°C for up to 6 hours. Images were taken with a Nikon T200 fluorescence microscope. The angiogenic index was calculated as the number of branch points in a visual field. Four different fields were counted for each treatment and the number of branch points was averaged. Error bars were calculated from means ± SD. For drug treatment experiments, cells were resuspended in medium containing 1 µM Cyclosporin A (Calcineurin inhibitor, Calbiochem) or 20 µM U73122 (PLCγ inhibitor, Calbiochem).

To investigate capillary tube formation in HUVECs harbouring the entire KSHV genome, HUVECs were infected with either a recombinant KSHV wildtype virus cloned in BAC (KSHVBACwt) or a K15 deletion mutant (KSHVBACΔK15) at an m.o.i. of 10 for 3 days to establish latency and 70–80% of GFP-expressing cells were obtained. In a similar way, HUVECs were infected with another recombinant KSHV virus (rKSHV.219) at an m.o.i of 10 for 72 hours and cells were then transfected with 100 pmol siRNA against K15 or control siRNA using the Neon transfection system. Twenty-four hours later the lytic cycle was activated with Na-Bu and a recombinant baculovirus expressing KSHV RTA (replication and transcription activator; see above) and 36 hours after induction of the lytic cycle, infected HUVECs were plated on matrigel and scored for capillary tube formation after 4–6 hours.

### Coimmunoprecipitation

One day prior to transfection, 4.2×10^6^ HEK293 cells were plated in a 10 cm dish. The cells were transiently transfected with retroviral vector plasmids pSF91-IRES-GFP, pSF91K15-IRES-GFP, pSF91K15/YF-IRES-GFP, pSF91K15/ΔSH3-IRES-GFP, pSF91K15/YF/ΔSH3-IRES-GFP (5 µg) using Fugene 6 transfection reagent (Promega) according to manufacturer's instructions. Forty-eight hours after transfection, cells were washed once in PBS and lysed in 500 µl of HEPES lysis buffer (20 mM HEPES, 150 mM NaCl, 0.5 mM EDTA, 1% Triton-X100, 1 mM DTT, 10% glycerol) containing protease and phosphatase inhibitors. 20 µl of the lysate was used as input control. Primary rabbit anti PLCγ1 (1∶50 dilution) was added to the rest of the lysate and incubated overnight with gentle shaking at 4°C. The next day the protein-antibody complexes were incubated with 20 µl of protein A Sepharose beads at 4°C with gentle shaking for 2–3 hours. Beads were washed three times with HEPES lysis buffer, and bound proteins were eluted and analyzed by Western blotting as described above, using a polyclonal antibody to K15. For co-immunoprecipitation in HEK293T cells, the expression vectors pFJ-K15P and pFJ-K15M [50], [52] were transfected with Fugene 6 transfection reagent and a polyclonal antibody to FLAG was used to detect the expression of K15.

### GST fusion protein binding assays

For GST pulldown experiments, *E. coli* Rosetta cultures transformed with GST-K15 expression or GST plasmids [50], [51] were grown at 37°C in LB-medium plus ampicillin and chloramphenicol. Cultures were induced at an optical density (600 nm) of 0.4–0.6 with 1 mM isopropyl-β-D-thiogalactopyranoside (IPTG) and harvested by centrifugation 5 hours after induction. Cell pellets were resuspended in 500 µl PBS with protease inhibitors, sonicated for 1 min on ice, supplemented with 1% Triton-X100 and incubated for 1 hour at 4°C. After centrifugation the supernatant was incubated with 100 µl glutathione sepharose beads (Amersham Biosciences) overnight at 4°C. Beads were washed twice with TBS-T containing protease inhibitors and run on an SDS polyacrylamide gel stained with Coomassie blue to estimate equal amounts of GST fusion proteins. HEK293T cells were washed once with PBS and lysed with TBS-T containing protease inhibitors for 10 min on ice. 100 µl of cleared lysates were incubated with pre-calculated amounts of either beads coated with GST fusion proteins or GST alone overnight at 4°C. Beads were washed three times with TBS-T containing protease inhibitors and analysed by SDS-PAGE and Western blotting.

### Statistics

Statistical analysis was performed using GraphPad Prism software. For the comparison of more than two groups a Kruskal-Wallis test with Dunn's post-test was applied. In the corresponding graphs, statistical significance was indicated with asterisks: p-value less than 0.05 (*), 0.01 (**), or 0.001 (***), (ns: not significant). Error bars were calculated from means ±SD.

## Results

### Identification of cellular genes induced by KSHV in a K15 dependent manner

In previous studies we had shown that, in epithelial cells, transiently transfected K15 induces the expression of chemokines, cytokines, anti-apoptotic genes and genes involved in signaling, inflammation as well as angiogenesis [51], [52], [58]. We next wanted to investigate a possible role of K15 in inflammation and/or angiogenesis in KSHV-infected primary endothelial cells, the origin of Kaposi's sarcoma. To this end, we compared the cellular transcriptome of primary human umbilical vein endothelial cells (HUVECs) infected with a recombinant KSHVwt, derived from the BAC36 genome [55], [56] or a K15 deletion mutant of BAC36, KSHVΔK15. HUVECs isolated from 2 donors were infected with recombinant KSHVwt or KSHVΔK15 at an approximate m.o.i. of 10 titered on HEK293, thus achieving an infection rate of approx. 70% in HUVECs as judged by the percentage of GFP expressing cells. As a control, the same amount of virus was heat-inactivated and used to mock-infect HUVECs. In parallel, HUVECs were also transduced with a retroviral vector expressing a codon optimized K15 cDNA [51].

Data were filtered for mRNAs showing an at least two-fold induction by K15 overexpression relative to empty vector transduced cells in each of two independent experiments performed. 199 appropriately annotated transcripts fulfilled the applied filter criteria. The top-ranking 50 transcripts were presented as a heatmap (figure 1A, lanes 1–2). In lanes 3 and 4, the relative expression in KSHVwt versus KSHVΔK15 infected cells is depicted for the same set of genes. Only six of the genes induced by overexpressed K15 (lanes 1–2) *i.e.* pro-melanin concentrating enzyme (PMCH), H2.0 like homeo-box (HLX1), nuclear receptor family 4, subfamily A, group 1 (NR4A1), Regulator of Calcineurin 1/Down syndrome critical region 1 (RCAN1/DSCR1), v-myc myelocytomatosis viral related oncogene (MYCN) and ndrg family member 1 (NDRG1) were found to be induced upon KSHVwt infection and were differentially regulated between KSHVwt- and KSHVΔK15-infected HUVECs in both experiments when a ratio of 2.0 was used as a cut off (figure 1A, lanes 3–4; genes indicated by arrows). Applying the same selection criteria to all 199 K15-induced genes revealed a total of eight genes, impaired in induction when K15 is deleted from the viral genome. In addition to those mentioned above, these are EH-domain containing 3 (EHD3) and Rho GTPase activating protein 25 (ARHGAP25) (not shown).

RCAN1/DSCR1 has been shown to play a role in angiogenesis in tissue culture based models of vascular tube formation [41], [59] and in transgenic models [44], [46]. Located in the genomic region duplicated in patients with Down syndrome, it is thought to affect tumor angiogenesis. Expression of RCAN1/DSCR1 is induced by VEGF-A through a pathway involving PLCγ1, Calcineurin and NFAT1 and it acts as a feedback modulator on Calcineurin [42]. We had previously noted its increased expression after transient transfection of a K15 expression vector in epithelial cells [52], [58] and therefore proceeded to investigate its role in KSHV infection of endothelial cells.

We first confirmed the upregulation of RCAN1/DSCR1 by K15 in endothelial cells using quantitative PCR and the samples tested by gene expression microarray. To confirm the expression of K15 in cells infected with KSHVwt and KSHVΔK15, we performed quantitative RT-PCR on the same RNA and could detect the expression of K15 in KSHVwt-infected cells but not in cells infected with KSHVΔK15 (figure 1B). Expression of K15 in HUVECs from a retroviral vector resulted in a marked increase in the mRNA levels of RCAN1 (15 and 19 fold in two experiments; see figure 1C), while in KSHVwt-infected cells we observed an increase of 3.2 and 6.4 fold relative to cells exposed to heat inactivated virus (figure 1D). In contrast, cells infected with KSHVΔK15 showed no increase in both independent experiments (1.1 fold and 1.0 fold) (figure 1D). As RCAN1/DSCR1 is known to be upregulated by vascular endothelial growth factor (VEGF-A) we explored if K15 also induces VEGF gene expression. Only a minimal (2.8-fold) increase of VEGF-A mRNA from a low constitutive level was detectable in one of the two microarray experiments, while the second experiment did not show any significant change (not shown). These data were confirmed by quantitative PCR (figure 1C). Furthermore, no change of VEGF-A mRNA expression in response to KSHVwt-infection was seen in microarrays and confirmatory qPCR results (figure 1D). These observations could suggest that K15 might induce RCAN1/DSCR1 expression in a VEGF-independent manner. We therefore explored a role of K15 in RCAN1/DSCR1 expression and KSHV mediated angiogenesis. RCAN1/DSCR1 will be referred to as RCAN1 from this point onwards.

### K15 induces angiogenic tube formation in an RCAN1-dependent manner

**Figure 2:**
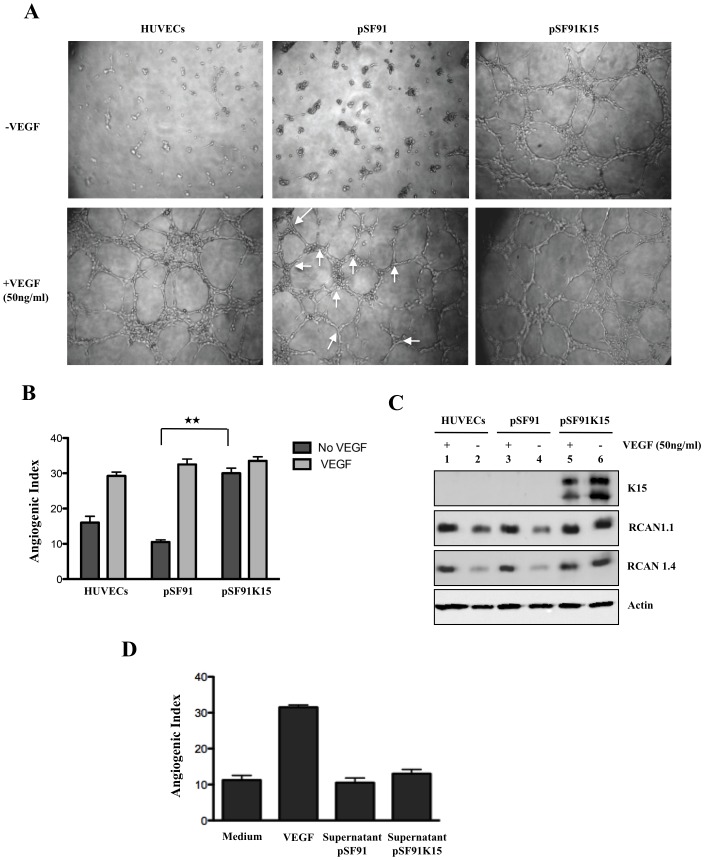
K15 induces capillary tube formation and increases RCAN1.1 and RCAN1.4 protein expression. (A) HUVECs transduced with pSF91 or pSF91K15 were plated on matrigel with medium (EBM2+2%FBS) with or without VEGF (50 ng/ml) 30 hours after transduction and were assessed 6 hours later for their ability to form capillary tubes. Arrows indicate cellular junctions counted to calculate the angiogenic index. (B) Angiogenic index (number of cellular junctions) in pSF91 and pSF91K15 transduced cells. Significance levels for the indicated comparisons are marked with ‘**’ (p<0.01) (see Material and Methods). (C) Western blot showing the increased expression of both RCAN1.1 and RCAN1.4 in K15 transduced cells in medium with and without VEGF. (D) HUVECs were resuspended in supernatant collected from cells transduced with K15 or vector control for 48 hours and then were plated onto matrigel for 6 hours to score for tube formation. Medium (EBM2+2%FBS) with VEGF (50 ng/ml) was used as a positive control.

**Figure 3:**
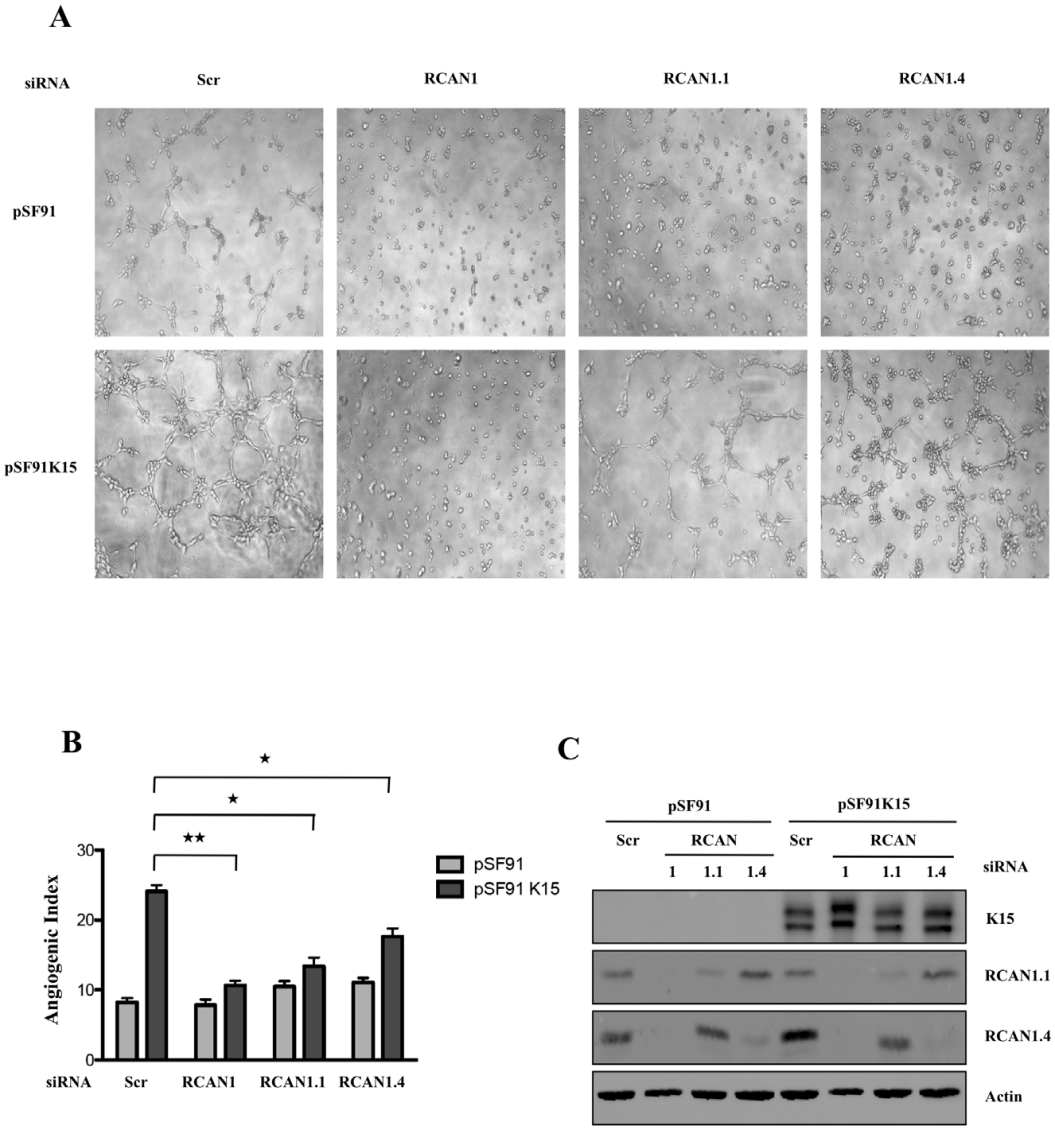
K15-induced capillary tube formation depends on RCAN1. HUVECs were transduced with pSF91K15 or the pSF91 vector and 30 hours later siRNAs against RCAN1, RCAN1.1 and RCAN 1.4 were transfected. 36 hours after transfection, cells were plated on matrigel to score for capillary tube formation (A, B) or analysed by Western blot to verify silencing of individual RCAN1 isoforms (C). Statistical significance levels for the comparisons of indicated samples are marked with ‘*’ (p<0.05) and ‘**’ (p<0.01).

**Figure 4:**
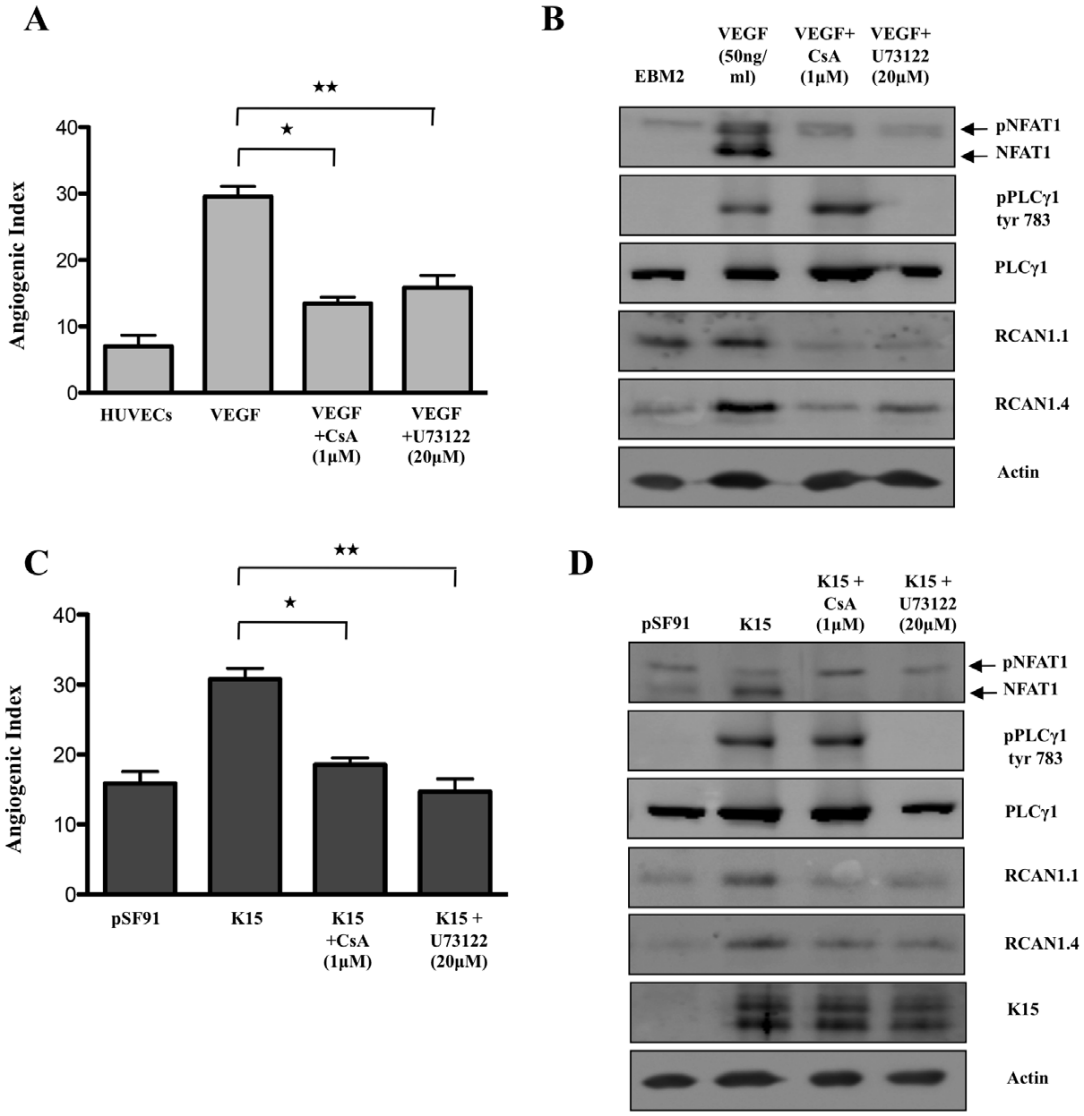
K15 dependent capillary tube formation involves the PLCγ1-Calcineurin-NFAT pathway. (A, B) HUVECs were treated with a Calcineurin inhibitor (Cyclosporin A; 1 µM), or a PLCγ inhibitor (U73122; 20 µM) for 6 hours and were then plated on matrigel in the presence of VEGF-A to score for capillary tube formation (A). Parallel samples were analysed on Western blots to show the phosphorylation of NFAT1 and PLCγ1 and the levels of total PLCγ1, RCAN1.1 and RCAN 1.4 (B). (C, D) HUVECs were transduced with pSF91 or pSF91K15 and after 30 hours were treated with CsA or U73122 and tube formation was analysed as in figure 2 (C). Phosphorylation of NFAT1, PLCγ1, and expression of RCAN1 isoforms and K15 was measured by Western blot (D).

**Figure 5:**
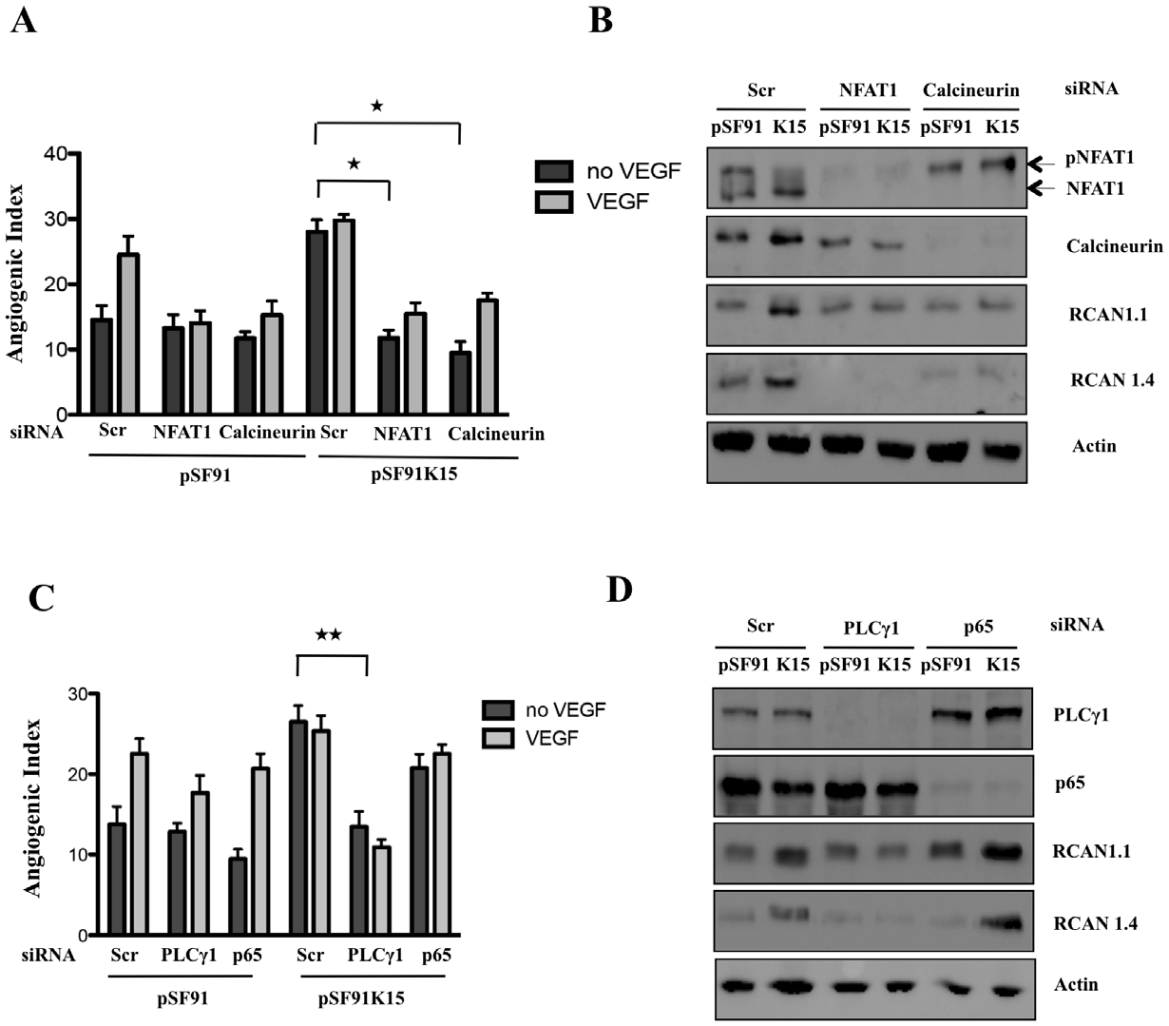
Silencing of Calcineurin, NFAT1 and PLCγ1 inhibits K15-induced capillary tube formation. HUVECs were transduced with pSF91 or pSF91K15 and (A) changes in the angiogenic index in response to K15 expression and silencing of NFAT1 or Calcineurin were analysed. (B) NFAT1 phosphorylation and expression of NFAT1, Calcineurin, RCAN1 in response to K15 overexpression and silencing of NFAT1 and Calcineurin in the absence of VEGF were analysed by Western blot. (C) Changes in the angiogenic index in response to K15 expression and silencing of PLCγ1 and NFκB p65. (D) Expression of RCAN1, PLCγ1, p65 in response to K15 overexpression and silencing of PLCγ1 or p65 in the absence of VEGF were analysed by Western blot. Transduction with pSF91, pSF91K15 and transfection with siRNA was performed as in figure 3.

**Figure 6:**
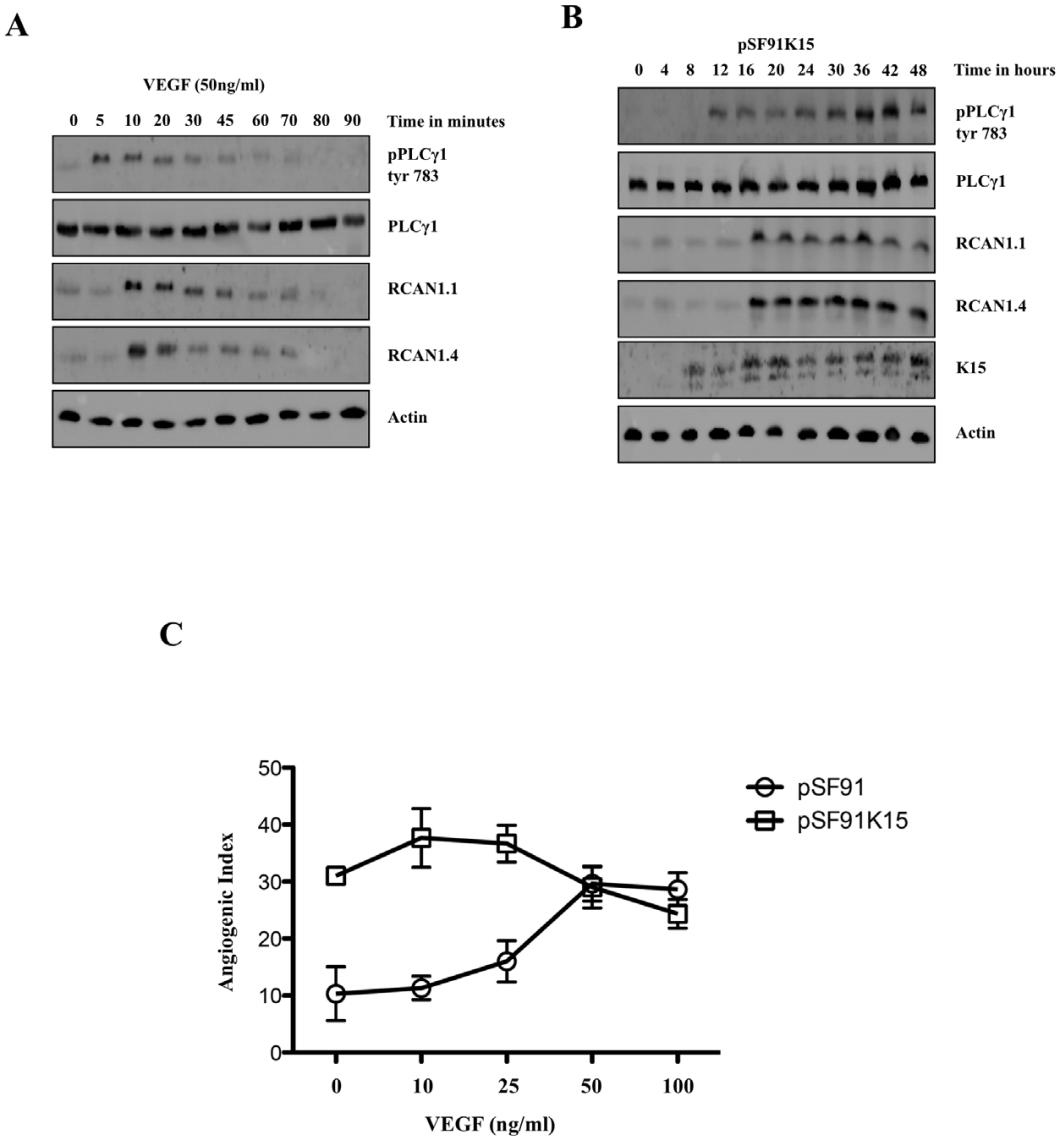
K15 activates PLCγ1 in a protracted manner via its SH2 binding domain. (A) HUVECs were treated with 50 ng/ml VEGF in 12 well plates for the indicated time points and analysed by Western blots to investigate the effects on the phosphorylation of PLCγ1 and expression of RCAN1 isoforms. (B) HUVECs were transduced with a retroviral vector expressing K15 (pSF91K15), plated in 12 well plates for the indicated time points and the phosphorylation of PLCγ1 and expression of RCAN1 isoforms was monitored by Western blot. (C) HUVECs were transduced with pSF91K15 or control vector and the angiogenic index was measured after treating the cells with VEGF at the indicated concentrations and plating them on matrigel.

As a measure of its angiogenic properties, VEGF can induce the branching of endothelial cells and formation of capillary ‘tubes’ in primary endothelial cells plated on matrigel [41], [59]. This is illustrated in the two left panels of figure 2A and can be quantitated using an angiogenic index, based on the number of branch points counted in 4 different fields (see figure 2, central panel, bottom row and Materials & Methods). To investigate a possible role of K15 in capillary tube formation, HUVECs were transduced with the retroviral vector encoding K15 (pSF91K15) or the control vector (pSF91) and plated on matrigel 30 hours after transduction in the absence or presence of 50 ng/ml VEGF. As compared to untransduced cells and the cells transduced with the control vector, cells expressing K15 could form tubes even in the absence of exogenously supplied VEGF (figure 2A) and the angiogenic index showed a statistically significant increase for K15-transduced cells relative to the control vector-transduced cells in the absence of VEGF (figure 2B, 3B, 4C, 5A, 5C, 6C, 7C). Western blot analysis (figure 2C) showed that stimulation of HUVEC with VEGF-A increases the levels of both the RCAN1.1 and RCAN1.4 protein isoforms (lane 1 and 3 relative to lane 2 and 4). K15, on the other hand, could upregulate both isoforms of RCAN1 even in the absence of exogenously supplied VEGF (lane 6 relative to lanes 2,4). These experimental results are in accordance with a role for K15 in angiogenesis that involves increased expression of RCAN1 isoforms.

**Figure 7:**
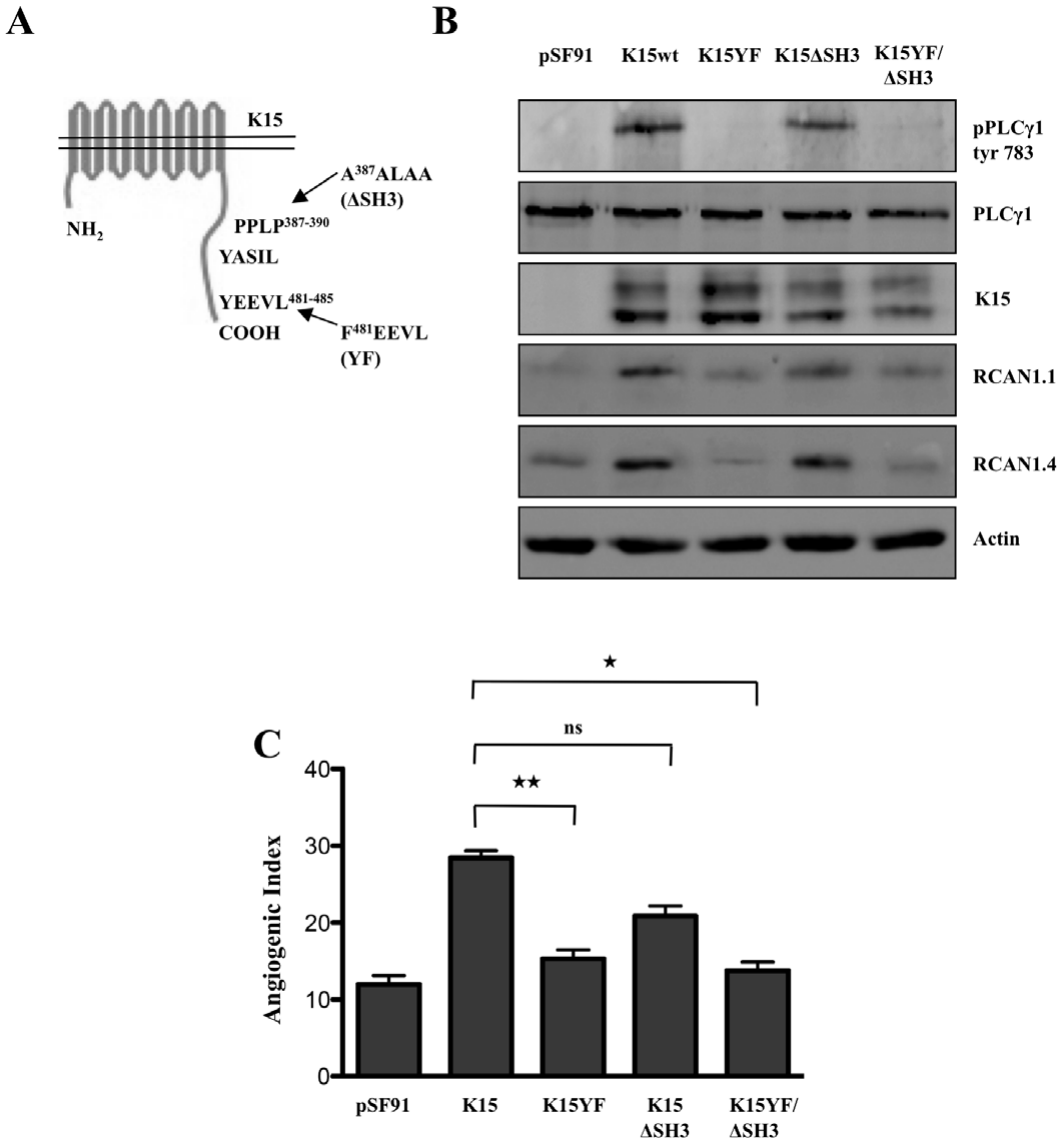
K15 activates PLCγ1 via its SH2 binding domain. (A) Schematic representation of K15 showing the SH2 and SH3 binding sites in the cytoplasmic domain along with corresponding mutants. (B) HUVECs were transduced with retroviral vectors expressing K15 and the indicated mutants of the K15 SH2 and SH3 binding sites or the control vector (pSF91). Thirty hours after transduction cells were lysed and analysed by Western blot for PLCγ1 phosphorylation and expression of K15 and RCAN1 isoforms. (C) Parallel samples from the experiment shown in (B) were plated on matrigel in the absence of VEGF and scored for capillary tube formation as in figure 2.

As other KSHV lytic proteins such as vGPCR and K1 have been reported to be involved in angiogenesis and to induce VEGF secretion [19], [20], [23], we next explored whether the induction of capillary tube formation by K15 could occur as a result of VEGF secretion. Fresh HUVECs were treated with supernatant from HUVECs transduced with the retroviral vector for K15 or the control vector and were then assessed for their ability to form capillary tubes on matrigel. While exogenously added VEGF induces capillary tube formation, conditioned supernatants from K15 or control vector, collected after 48 hours, did not (figure 2D). Taken together with the fact that expression of K15 in primary endothelial cells does not consistently induce expression of the VEGF-A gene (figure 1), this observation is compatible with the interpretation that K15 does not increase RCAN1 expression nor induces capillary tube formation by augmenting VEGF secretion. Additional evidence in support of this interpretation is provided below. This experiment does not exclude other paracrine effects mediated by K15, e.g. as a result of its induction of IL-8 secretion [52], [58].

To further investigate if the K15-induced capillary tube formation in the absence of VEGF requires RCAN1 expression, we targeted RCAN1 expression in both control vector and K15-transduced cells using a pool of RCAN1 siRNA that recognizes all the known isoforms of RCAN1. In HUVECs transduced with a GFP expressing retroviral vector pSF91, knockdown of RCAN1 significantly abrogates VEGF-induced tube formation in contrast to cells transfected with a scrambled siRNA (not shown) in line with earlier reports that RCAN1 is required for VEGF-induced capillary tube formation in HUVECs [41], [42]. Knockdown of RCAN1 also abrogated K15-induced capillary tube formation in the absence of VEGF (figure 3A and 3B). The Western blot in figure 3C confirms the knockdown of RCAN1. We next selectively abrogated the expression of RCAN1 isoforms, RCAN1.1 and RCAN1.4, with specific siRNA. As shown in figure 3A–C, expression of RCAN1.1 and RCAN 1.4 could be reduced selectively, and the absence of either isoform reduced the ability of K15 to induce angiogenic tube formation. Together, these experiments suggest that K15 plays an important role in the induction of capillary tube formation that might be dependent on both RCAN1 isoforms.

### K15 activates PLCγ1 and upregulates RCAN1 via the Calcineurin-NFAT1 pathway

Previous reports have shown that VEGF-A regulates RCAN1.4 expression by activation of the classical Ca^2+^/Calcineurin pathway leading to activation of the transcription factor NFAT in endothelial cells [41]. Following VEGF-A stimulation, phosphorylation of the tyrosine 1175 site in VEGFR-2 allows the binding and subsequent phosphorylation of PLCγ1. PLCγ1 is then able to catalyze the hydrolysis of the membrane phospholipid phosphatidylinositol (4,5)-bisphosphate (PIP2) resulting in the generation of diacylglycerol (DAG) and inositol 1,4,5-trisphosphate (IP3). DAG is a physiological activator of PKC, whilst IP3 acts upon the endoplasmic reticulum to release calcium, thus inducing a rise in intracellular calcium (Ca^2+^). Increased Ca^2+^ levels activate the phosphatase Calcineurin, which dephosphorylates members of NFAT family. This dephosphorylation allows the translocation of NFAT into the nucleus, where it binds to NFAT consensus sequences present in the promoter region of several genes involved in angiogenesis and cellular migration, such as RCAN1 or COX-2, resulting in their increased expression. Since our findings so far suggest that K15 mimics the effect of VEGF in inducing the expression of RCAN1 and increasing capillary tube formation, and as we had observed earlier that K15 activates an NFAT-responsive promoter [52], we further investigated whether the Calcineurin-NFAT pathway was involved in the upregulation of RCAN1.

As expected from previous reports [42], [59], we could inhibit VEGF-induced capillary tube formation in HUVEC (figure 4A), NFAT1 dephosphorylation and the resulting increased RCAN1.1/1.4 expression (figure 4B) with chemical inhibitors of Calcineurin (cyclosporin A; CsA) and PLCγ (U73122). In addition, as shown in figure 4C, K15-induced capillary tube formation was significantly abrogated in the presence of CsA and U73122, suggesting that it follows the same pathway as VEGF. Western blot analysis (figure 4D) showed that K15 induced PLCγ1 phosphorylation and dephosphorylation of NFAT1. Treatment with CsA and U73122 reduced the levels of K15-induced RCAN1.1/1.4 expression, abolished dephosphorylation of NFAT1 and (in the case of U73122) reduced levels of PLCγ1 phosphorylated on Tyr 783.

To further investigate the involvement of Calcineurin, NFAT1 and PLCγ1 in K15-induced tube formation we targeted their expression using siRNA. As shown in figure 5A and 5B, suppressing protein levels of Calcineurin and NFAT1 significantly abrogated angiogenic tube formation induced by VEGF, as well as by K15 in the absence of VEGF. Western blots of the same samples (figure 5B) showed again that K15 expression led to an enhanced dephosphorylation of NFAT1 and that this effect was abolished by silencing the expression of Calcineurin by means of siRNA. Silencing of NFAT1 and Calcineurin also reduced K15-induced RCAN1.1 and RCAN1.4 levels (figure 5B).

Having observed that expression of K15 results in an increased phosphorylation of PLCγ1 and that a chemical inhibitor of PLCγ (U73122) reduced K15-induced capillary tube formation (figure 4C and 4D), we next examined if silencing of PLCγ1 by siRNA would also antagonize these effects of K15. As shown in figure 5C, knockdown of PLCγ1 decreased VEGF-induced capillary tube formation in the absence of K15 (pSF91-transduced cells in figure 5C), as well as K15-induced capillary tube formation (figure 5C) and RCAN1.1/1.4 expression (figure 5D) in the absence of VEGF.

We have shown earlier that K15, through its cytoplasmic C terminal domain activates the NFκB pathway [52], [58], [60] and that this involves the NFκB component RelA/p65 (unpublished). To investigate the involvement of the NFκB pathway in K15-induced capillary tube formation, we silenced RelA/p65 using siRNA. As shown in figure 5C and 5D, silencing of RelA/p65 does not antagonize RCAN1 expression and capillary tube formation induced by K15. Taken together, these results suggest that the activation of the PLCγ1-Calcineurin-NFAT pathway is required for the K15-induced tube formation and that this effect is independent of the NFκB pathway.

### K15 activates the PLCγ1 pathway and upregulates expression of RCAN1 in a protracted manner

We next studied the kinetics of the activation of the PLCγ1 pathway by K15. We found that, in contrast to VEGF, which induces a transient increase in PLCγ1 phosphorylation that peaks at 5–10 minutes and disappears after approx 30 minutes (figure 6A), the K15-induced PLCγ1 phosphorylation lasted for up to 48 hours (figure 6B). Treatment with VEGF results in a rapid increase of RCAN1.1/1.4 protein levels, which however decrease after 30 minutes, despite the continuous presence of VEGF in this experiment. This suggests a transient VEGF-dependent signaling and rapid turnover of RCAN1 proteins (figure 6A). In contrast, in K15-transduced cells RCAN1.1/1.4 levels increased in parallel with K15 protein levels and remained high, along with levels of phosphorylated PLCγ1, for the duration of this experiment (48 hours) (figure 6B). These results indicate that VEGF induces a short pulse of PLCγ1 activation, which leads to a transient expression of RCAN1. K15, on the other hand, as seen in figure 6B, activates PLCγ1 and RCAN1 expression in a protracted constitutive manner.

We also explored if the effects of VEGF and K15 on capillary tube formation are additive. As shown in figure 6C, increasing concentrations of VEGF led to a gradual increase in angiogenic tube formation in vector-transduced, but not in K15-transduced cells. This observation suggests that, in K15 expressing endothelial cells, the PLCγ1-Calcineurin-NFAT pathway is maximally activated and cannot be further stimulated by exogenous VEGF.

### The SH2 binding motif of K15 is important for the activation of PLCγ1 and capillary tube formation

As reported earlier, K15 is a trans-membrane protein containing eight predicted trans-membrane regions and a C- terminal cytoplasmic domain [47], [48], [50], [52], [58]. The cytoplasmic domain of the P-type of K15 used here contains two SH2-binding sites (Y^431^ASIL and Y^481^EEVL), a proline-rich SH3-binding site (P^387^PLP) and a TRAF-binding site (A^473^TQPTDD) [50], [52]. The Y^481^EEVL motif has been shown to activate various signaling pathways such as MAPK, JNK, NFκB [47], [50], [52]. To test which of these domains is involved in the activation of the PLCγ1 pathway, we used previously reported mutants of SH2 and SH3 binding motifs [51] in which the tyrosine at position 481 had been changed to phenylalanine (YEEVL to FEEVL), or all the prolines in the SH3 binding motif had been changed to alanine (PPLP to AALA). We also employed a double mutant in which both of these domains had been mutated [51] (figure 7A). We tested these mutants for their ability to induce capillary tube formation and to activate PLCγ1 phosphorylation. As shown in figure 7B, wildtype K15 and the ΔSH3 mutant could induce PLCγ1 phosphorylation and increase RCAN1.1/1.4 expression, whereas the tyrosine 481 (YF) mutant of K15 could not. As shown in figure 7C, mutants K15/YF and K15/YF/ΔSH3 could not induce capillary tube formation, while mutant K15ΔSH3 showed only a moderately (not significantly) lower angiogenic index than K15wt. This observation suggests that the tyrosine residue at position 481 in the YEEV motif is important for activation of the PLCγ1 pathway, induction of RCAN1 and capillary tube formation.

### Both K15P and K15M isoforms directly interact with and activate PLCγ1

**Figure 8:**
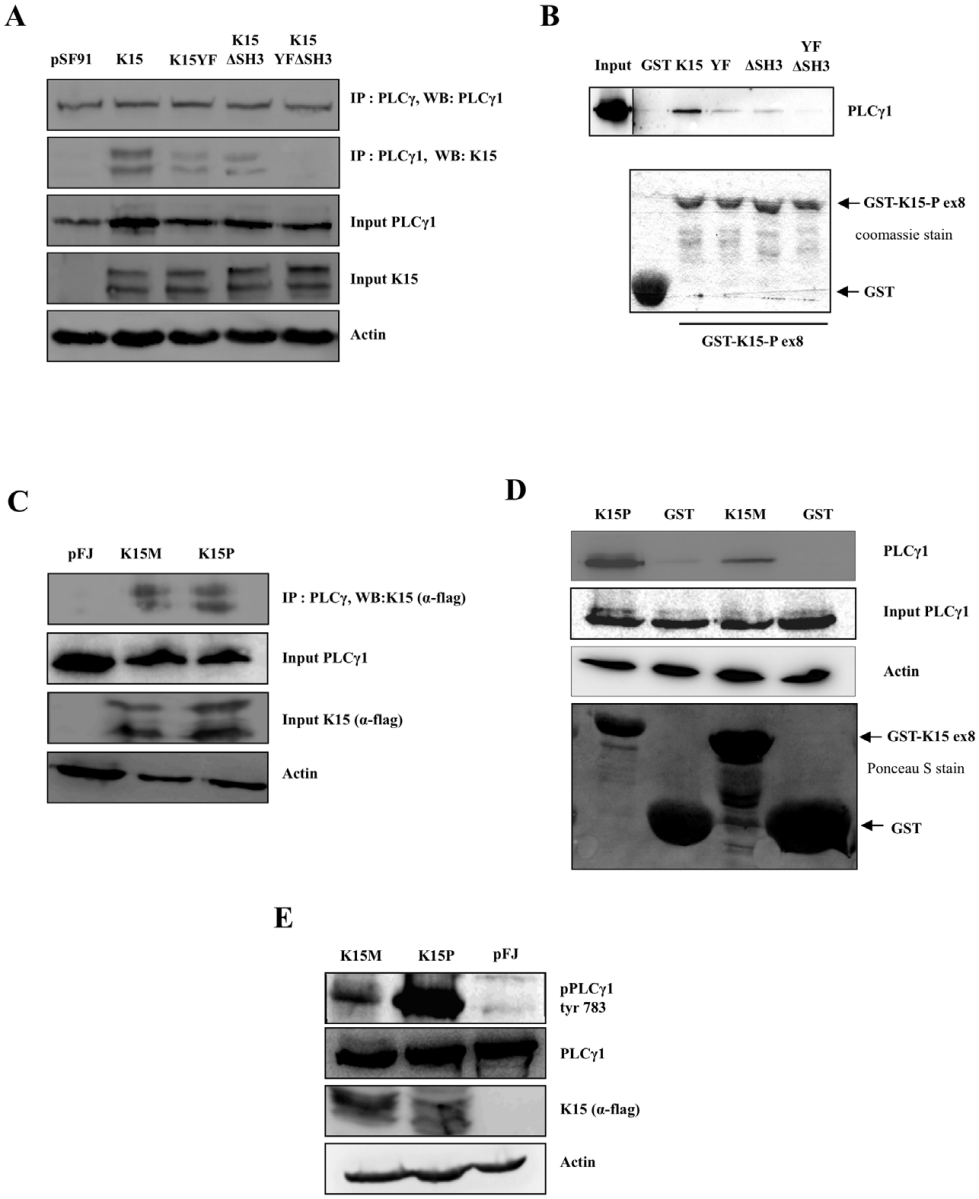
K15P and K15M recruit and activate PLCγ1. (A) Co-immunoprecipitation of K15-P with PLCγ1: pSF91K15, the mutants (YF, ΔSH3, YF/ΔSH3) and control vector (pSF91) were used to transiently transfect HEK293. After immunoprecipitation of endogenous PLCγ1, co-immunoprecipitated K15-P was visualised on Western blots using a polyclonal antibody to K15. (B) Purified GST or GST fusion proteins containing the cytoplasmic domain of K15-P wt and mutants YF, ΔSH3, YF/ΔSH3 were used in a GST pulldown assay with lysates of HEK293T cells. Endogenous PLCγ1 bound to GST-K15 beads was visualised with an antibody against PLCγ1. Purified GST or GST-K15 fusion proteins were visualized by Coomassie blue staining to show that equal amounts of fusion protein were used for GST pulldown experiments (bottom). (C) Co-immunoprecipitation of K15-P and K15-M with PLCγ1: HEK293T cells were transiently transfected with a vector containing a FLAG-tagged K15P or K15M, or the control vector (pFJ) and endogenous PLCγ1 was immunoprecipitated. Co-immunoprecipitated K15-P or K15-M was visualised by an antibody to the FLAG tag. (D) Lysates of HEK293T cells were incubated with beads coated with GST, GST-K15-P or GST-K15-M and bound endogenous PLCγ1 was detected on Western blots. (E) Lysates of HEK293T cells transfected with K15-P or K15-M expression vector or control vector (pFJ) were analysed on Western blots using an antibody to the PLCγ1 tyrosine 783 phosphorylation site.

VEGF-induced activation of PLCγ1 in endothelial cells involves recruitment of PLCγ1 to the phosphorylated cytoplasmic tail (on tyrosine 1175) of VEGFR2 and subsequent phosphorylation of PLCγ1 on tyrosine 783 [39]. Since we had found that activation of PLCγ1 by K15 requires the K15 Y481 residue, which had been previously shown to be phosphorylated by Src family tyrosine kinases [47], [52], we next investigated if K15 can directly recruit PLCγ1 via its Y^481^EEV SH2 binding site. To test this hypothesis, we performed co-immunoprecipitation experiments in HUVECs transduced with K15 and control vector. Endogenous PLCγ1 was immunoprecipitated with an antibody to PLCγ1 and immunoprecipitates were probed on Western blots with an antibody for K15. We found that K15 can interact with PLCγ1 (figure 8A), and that the K15YF mutant and the K15ΔSH3 mutants showed a reduced interaction with PLCγ1. The K15 double mutant (K15YF/ΔSH3) showed no interaction. This suggests that K15 interacts with PLCγ1, and that the SH2 binding site (Y^481^EEV) as well as the SH3 binding site (P^387^LPP) contribute to the interaction. The interaction between the cytoplasmic domain of K15 and endogenous PLCγ1 was also confirmed in a GST pull-down assay using the cytoplasmic domain of K15 fused to GST (figure 8B). In this assay, the Y481F and ΔSH3 mutants also showed a decreased interaction while virtually no interaction could be seen in the case of the double mutant (YFΔSH3).

The K15 gene occurs in several allelic isoforms, thought to originate from recombination events with related rhadinoviruses [48], [49], [58]. In the experiments described so far, we had used the ‘predominant’ P allele of K15 (K15-P). To explore if other K15 isoforms also recruit PLCγ1, we tested the most divergent isoform, the M-type of K15 (K15-M). As shown in figure 8C, D, both K15-P and K15-M co-immunoprecipitate with PLCγ1 and interact with PLCγ1 in a GST pull-down assay. In addition, transfection of both K15-P and K15-M induces phosphorylation of PLCγ1 on tyrosine 783 (figure 8E). As K15-M is more divergent from K15-P than other isoforms [61], it is likely that all K15 isoforms are able to activate PLCγ1.

### The activation of PLCγ1 by K15 is independent of VEGFR2 and Src kinases

**Figure 9:**
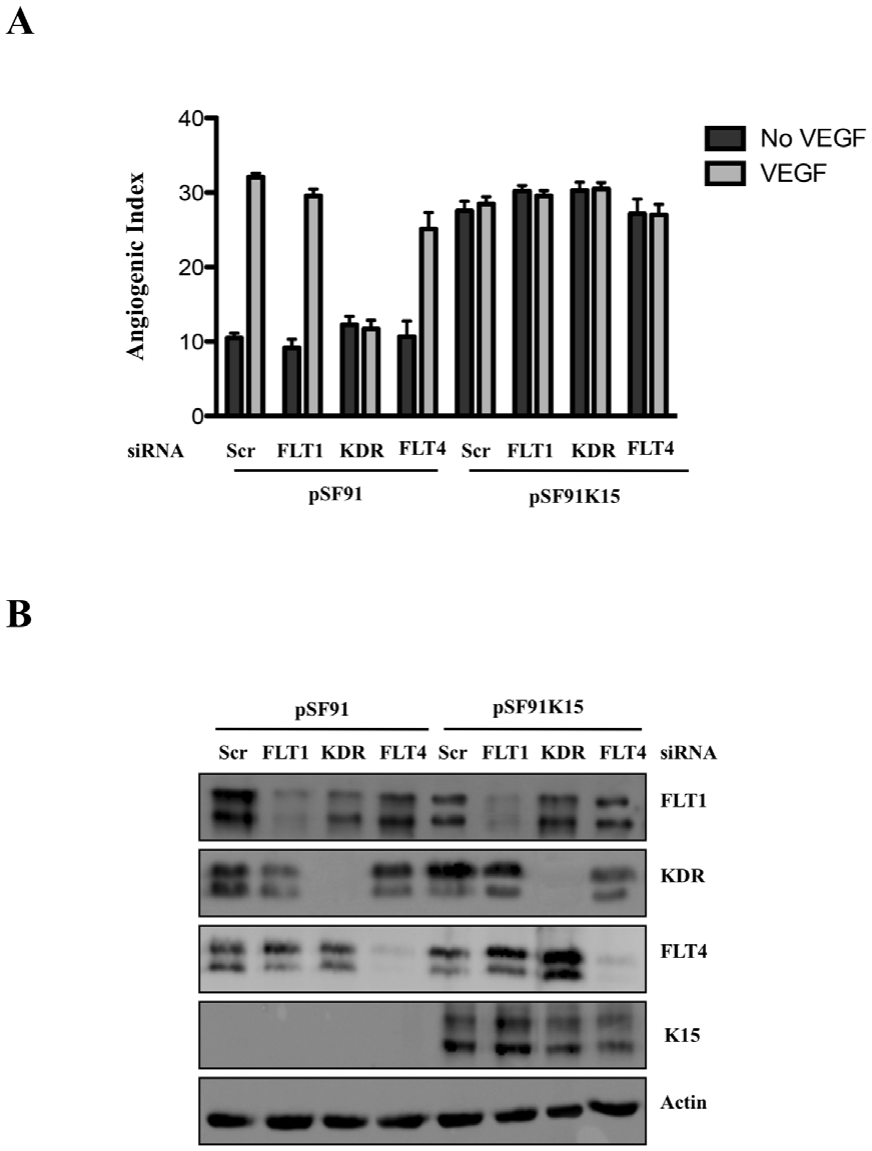
K15 induced angiogenesis does not involve VEGF receptors. (A) HUVECs were transduced with pSF91 or pSF91K15 and 30 hours later transfected with siRNA to VEGFR1 (FLT1), VEGFR2 (KDR) and VEGFR3 (FLT4). Thirty-six hours after transfection, cells were plated on matrigel in the absence or presence of VEGF and scored for capillary tube formation. (B) Parallel samples were analysed by Western blot to verify silencing of VEGF receptors and K15 expression.

To exclude that low levels of VEGF might contribute to K15-dependent PLCγ1 activation by activating VEGF receptors that could, in turn, phosphorylate PLCγ1, we used siRNA to silence the expression of all three VEGF receptors. As shown in figure 9A, capillary tube formation in response to VEGF treatment is completely abrogated when the expression of KDR/VEGFR2 is silenced in HUVECs transduced with the parental retroviral vector, while no significant reduction is seen following silencing of the other two VEGF receptors, FLT1/VEGFR1 and FLT4/VEGFR3. This is in line with previous reports that VEGFR2 (KDR) is the prime receptor for VEGF-induced pathways in HUVECs [40]. In contrast, in K15-transduced cells, the K15-induced capillary tube formation in the absence of VEGF is not reduced when KDR/VEGFR2 is silenced suggesting that the K15 mediated activation of PLCγ1 is independent of VEGFR2. Likewise, neither FLT1/VEGFR1 nor FLT4/VEGFR3 appear to be involved in K15-induced capillary tube formation (figure 9A, B). This supports our previous result (figure 1C, 2D and 6C) that K15-induced tube formation is independent of VEGF.

To explore if Src family kinase members are involved in the K15-mediated activation of PLCγ1, we treated K15-transduced HUVECs with the Src family kinase inhibitors Su6656, PP1, PP2 and MNS. We could not observe a strong reduction of K15-induced PLCγ1 phosphorylation or angiogenic tube formation in these experiments (data not shown). The identity of the tyrosine kinase involved in the phosphorylation of K15 tyrosine 481 (required for PLCγ1 docking) or PLCγ1 tyrosine 783 therefore remains to be established.

### Lack of K15 abrogates KSHV-induced capillary tube formation

**Figure 10:**
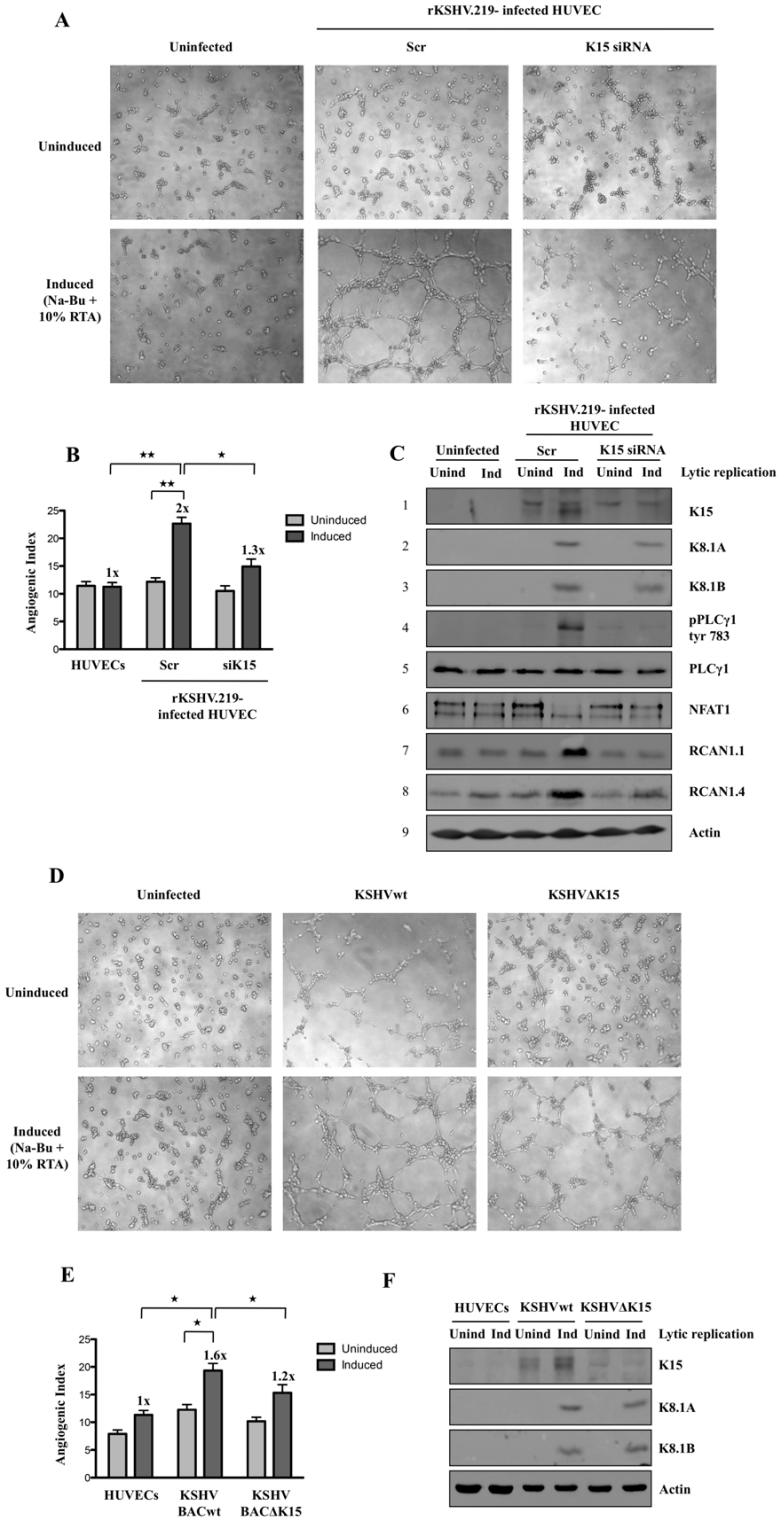
Lack of K15 abrogates angiogenic tube formation induced by KSHV infection of HUVECs. (A–C) Capillary tube formation in HUVECs infected with rKSHV.219. Seventy-two hours after infection of HUVECs, siRNA against K15 or control siRNA was microporated. Twenty-four hours after transfection the lytic cycle was induced with sodium butyrate (Na-Bu) and 10% RTA. Forty-eight hours later, cells were plated on matrigel and scored for tube formation (A). (B) Angiogenic index for the experiment shown in (A). Significance levels for the indicated comparisons of different samples are marked with ‘*’ (p<0.05) and ‘**’ (p<0.01). (C) Western blot analysis for the experiment shown in (A) to confirm the silencing of K15 expression and to quantify the expression of K8.1 (a late lytic glycoprotein), RCAN1.1/1.4, and phosphorylated PLCγ1 and phosphorylated NFAT1. (D–F) HUVEC were infected with BAC-derived KSHVwt or KSHVΔK15 for 72 hours. Following activation of the lytic cycle as in (A–C), cells were plated on matrigel for angiogenic tube formation (D). (E) Angiogenic index for the experiment shown in (D). Significance levels for the indicated comparisons are marked with ‘*’ (p<0.05). (F) Parallel samples were analysed by Western blot for the expression of K15 and K8.1 A/B.

Infection of primary endothelial cells with KSHV has previously been shown to induce the formation of capillary tubes when infected cells are plated on matrigel. Several other KSHV proteins including K1, vGPCR, and vIL6 have been reported to have angiogenic effects and/or induce the secretion of VEGF [19], [20], [25], [29]. As we had found that the increased expression of RCAN1, a mediator of VEGF-mediated angiogenesis [41], in KSHV-infected cells depends on the presence of K15 in the viral genome (figure 1), we next explored if K15 contributes to the angiogenic properties of KSHV in KSHV-infected cells. We infected HUVECs with a recombinant KSHV virus (rKSHV.219) for 3 days to obtain 70–80% GFP expressing cells before microporating these cells with siRNA against K15. Twenty-four hours later the lytic replication cycle was activated with Na-Bu and a recombinant baculovirus expressing KSHV RTA (regulator of transcriptional activator) and 36 hours after activation of the lytic cycle, infected HUVECs were plated on matrigel and scored for capillary tube formation after 6 hours. As shown in figure 10A, capillary tube formation is visible in cells transfected with control siRNA following the induction of the lytic cycle. In contrast, angiogenic tube formation is reduced upon silencing of K15, suggesting a role of K15 in virus-induced angiogenesis. Figure 10B shows that both the increase of the angiogenic index upon activation of the lytic cycle, as well as its decrease upon silencing of K15, are statistically significant. The top panel of figure 10C shows the effective silencing of K15 in this experiment and its increased expression following induction of the lytic cycle in cells treated with control siRNA. As reported previously [52], the upper band seen in the western blot is not affected by K15 siRNA, while the lower band represents the 43 kDa K15 protein seen in KSHV infected cells. Expression of the two isoforms of the KSHV structural K8.1 glycoprotein is used as a marker for lytic cycle induction (panels 2,3 of figure 10C). As shown in panel 4 of figure 10C, induction of the lytic replication cycle results in increased PLCγ1 phosphorylation, which, along with capillary tube formation, is strongly suppressed by silencing of K15. In addition, activation of the lytic replication cycle and increased PLCγ1 phosphorylation is accompanied by NFAT1 dephosphorylation and increased levels of RCAN1.1 and RCAN1.4, which are reversed by silencing of K15.

In an additional experiment, we compared the ability of BAC-derived KSHVwt and KSHVΔK15 to induce angiogenic tube formation. We infected HUVEC with cell-free KSHVwt and KSHVΔK15 to obtain approximately 60% of GFP expressing cells. As shown in figure 10 D, E, BAC-derived KSHV induced moderate angiogenic tube formation in latently infected compared to uninfected cells, and more extensive angiogenic tube formation following activation of the lytic cycle, whereas the angiogenic index was reduced in KSHVΔK15- relative to KSHVwt-infected lytically induced cells. Western blots (figure 10F) confirmed the absence of K15 protein in KSHVΔK15- compared to KSHVwt-infected cells (top panel) and comparable levels of lytic glycoprotein K8.1 expression in KSHVwt and KSHVΔK15-infected cells following lytic cycle activation. These results suggest that the increased formation of capillary tubes that occurs in KSHV infected endothelial cells depends to a significant extent on K15 and the K15-dependent activation of the PLCγ1-NFAT1-RCAN1 pathway.

## Discussion

Kaposi's Sarcoma Herpesvirus is the cause of Kaposi's Sarcoma (KS), a vascular tumor with pronounced inflammatory histological features that arises from infected endothelial cells. Early stages of KS (“patch, plaque”) are characterized by inflammatory infiltration and features of aberrant angiogenesis [4], [5], [62]. In advanced lesions (nodular KS), KSHV-infected endothelial spindle cells predominate and are thought to represent the neoplastic component of this tumor [4], [5], [62]. Unlike most cancers, KS represents, in most cases, an oligo- or polyclonal proliferation of infected endothelial cells [63], [64], suggesting that independent infection events, rather than the spread of a clonal tumor are responsible for the dissemination of KS to different organs and body sites [63], [64]. KS lesions may regress following the treatment of HIV with anti-retroviral therapy, or moderating iatrogenic immune suppression (in transplant recipients), indicating that this tumor requires the continued presence of KSHV and the expression of viral genes. Thus KS resembles other virus-driven proliferative diseases such as, in particular, EBV-associated post-transplant lymphoproliferative disease (PTLD) (reviewed in [4], [5], [62]).

KSHV induces a transcriptional reprogramming of vascular endothelial cells towards a pattern typical of lymphatic endothelial cells, as well as inducing cellular genes typical of vascular endothelial cells in infected lymphatic endothelial cells [15]–[17] In addition, KSHV causes endothelial to mesenchymal transition (EndoMT) in infected lymphatic endothelial cells, thereby accounting for the expression of mesenchymal markers frequently observed on KS spindle cells [65]. Both the latent viral protein vFLIP and the lytic protein vGPCR appear to contribute to this phenotype [65]. In cultured primary endothelial cells, KSHV can induce the formation of capillary junctions when infected cells are plated on matrigel [18]. It is likely that the KSHV-induced formation of capillary junctions may explain the aberrant angiogenesis seen in KS lesions, rather than the formation of spindle cells, which is mainly due to the latent vFLIP protein [13], [14].

In this study we wanted to define a possible role of the KSHV K15 protein in KSHV infected spindle cells. Starting from an earlier observation [52], that transfection of K15 into epithelial cells induced the expression of cellular genes that are known to be activated by VEGF, but did not appear to induce VEGF expression itself, we compared the effect of expressing K15 in primary endothelial cells on cellular gene expression with the pattern of cellular genes induced by infecting endothelial cells with KSHV, or a KSHV K15 deletion mutant. Only a small subset of cellular genes that can be induced by over-expressed K15 appear to be differentially regulated in KSHVwt- and KSHVΔK15-infected endothelial cells (figure 1). This likely reflects the fact that K15 is only weakly expressed in infected endothelial cells prior to the activation of the lytic cycle (figure 10F) and is reminiscent of our recent observation that only a small selection of cellular genes activated by over-expressed vFLIP are measurably increased in endothelial cells infected with KSHVwt compared to a deletion mutant of vFLIP, KSHVΔvFLIP [14].

Cellular genes that are differentially regulated between endothelial cells infected with KSHVwt and a KSHV deletion mutant are likely to be those whose expression is most strongly affected by the deleted viral gene in the context of the entire virus genome. In this study we found RCAN1/DSCR1, a regulator of the Calcineurin-NFAT pathway, to be differentially expressed in endothelial cells infected with KSHVwt and KSHVΔK15. The activation of RCAN1 by K15 in KSHVwt- but not in KSHVΔK15-infected endothelial cells, as well as in endothelial cells transduced with K15, is not accompanied by a consistently increased expression of VEGF family members (figure 1A–D).

Expression of RCAN1 is normally induced by VEGF (figure 2C) and is required for VEGF-induced angiogenic tube formation (figure 2A,B). Similarly, RCAN1 expression is required for K15-induced angiogenesis in the absence of exogenously added VEGF (figure 3B,C). Although the expression of RCAN1 is normally regulated by the binding of VEGF to its receptor, the subsequent activation (phosphorylation) of PLCγ1 and the ensuing influx of Ca^2+^, our observations suggest that the K15-induced expression of RCAN1 and angiogenic tube formation is not dependent on VEGF. The expression of VEGF-A is not consistently increased by overexpressed K15 whereas, the expression of RCAN1 is (figure 1C). Conditioned supernatants from K15 transduced cells do not induce the angiogenic tube formation caused by VEGF (figure 2D) and the silencing of any of the three VEGF receptors FLT1, KDR, FLT4 by siRNA does not reduce K15-induced angiogenic tube formation while VEGF-induced angiogenesis is inhibited by silencing KDR (figure 9A,B). We therefore conclude that K15-induced angiogenesis is independent of VEGF and VEGF receptors, in spite of K15-induced angiogenesis requiring several of the downstream components of the VEGF signaling pathway.

Like VEGF, expression of K15 induces the dephosphorylation of NFAT (figure 4) and this requires PLCγ1 and Calcineurin, since small molecule inhibition of PLCγ1 (U73122) and Calcineurin (cyclosporin A) (figure 4), as well as the siRNA-mediated silencing of PLCγ1, Calcineurin and NFAT1 (figure 5) inhibit K15-induced RCAN1 expression and K15-dependent angiogenesis. Both RCAN1 isoforms RCAN1.1 and RCAN1.4 appear to contribute to K15-induced angiogenic tube formation. These results suggest that K15 activates a PLCγ1-Calcineurin-NFAT1-dependent pathway, normally utilized by VEGF. In contrast to VEGF, which induces a short burst of PLCγ1 phosphorylation and a transient RCAN1 expression, K15 activates PLCγ1 phosphorylation and RCAN1 expression in a protracted manner (figure 6). Whether this is due to K15 not being subject to the same negative feedback regulation that operates in the case of VEGFR-dependent signaling, or to other mechanisms, remains to be established. Whatever the molecular basis of this observation, a constitutive activation of PLCγ1 by K15 could contribute to the sustained angiogenic phenotype seen in KS lesions. In K15-expressing cells, the PLCγ1-Calcineurin-NFAT pathway seems to be fully activated and cannot be further enhanced by exogenous VEGF, in keeping with the interpretation that K15 and VEGF use the same pathway.

In an attempt to understand how K15 would achieve the activation of this VEGF-dependent pathway without involving VEGF or VEGF receptors, we found that K15, a membrane protein, can directly interact with PLCγ1 (figure 8A,B). Both allelic isoforms of K15, K15-P and K15-M, are able to recruit and activate PLCγ1 (figure 8C–E), suggesting that KSHV isolates with divergent K15 variants are able to activate angiogenesis through the mechanism described in this report. An SH2 binding site in the cytoplasmic domain of K15, YEEVL, is required for K15-induced phosphorylation of PLCγ1 (figure 7B), and this site, as well as an SH3-binding site in the same cytoplasmic K15 region, contributes to the recruitment of PLCγ1 to K15 (figure 8A,B). It is therefore conceivable, but remains to be shown, that SH2 and SH3 domains, as they occur in the γ specific array of PLCγ1, may be involved in the recruitment of PLCγ1 to K15. Unlike the VEGF receptors, K15 is not known to have tyrosine kinase activity. Since silencing of VEGF receptors does not affect K15-induced angiogenesis and since several Src kinase inhibitors did not show a strong effect on K15-induced PLCγ1 phosphorylation and angiogenic tube formation (data not shown), the phosphorylation of PLCγ1 recruited to K15 most likely involves another, yet to be determined, tyrosine kinase.

**Figure 11:**
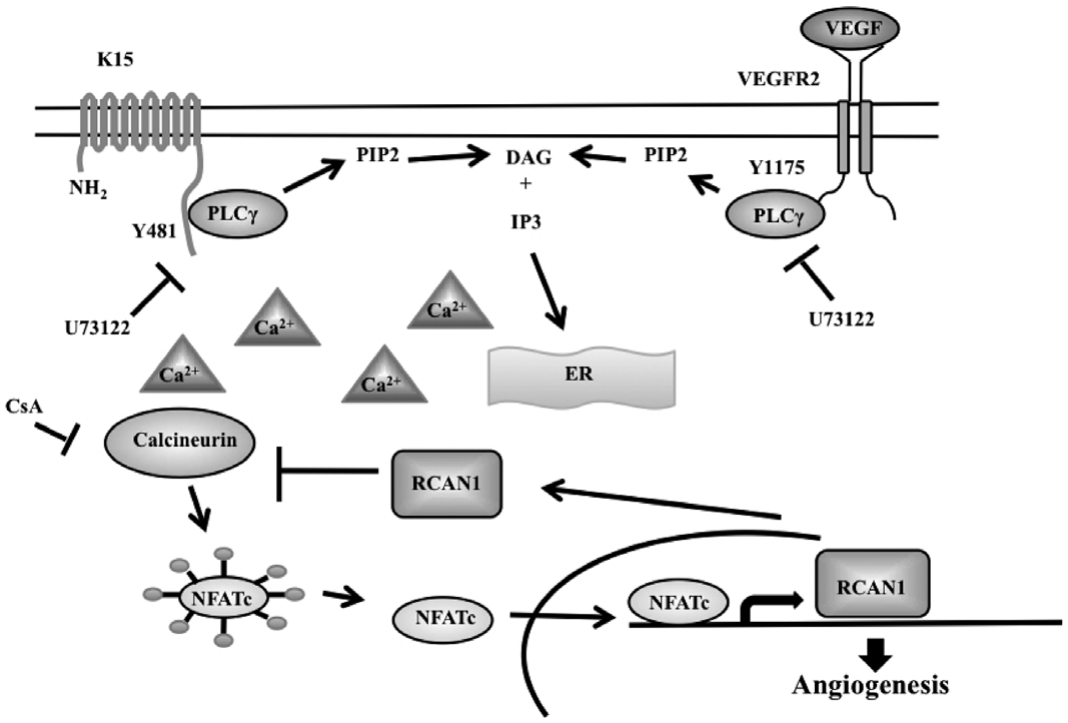
Schematic diagram showing the likely involvement of K15 in the PLCγ1-Calcineurin-NFAT pathway. As shown in this report, K15 directly recruits PLCγ1 and induces its phosphorylation by an unidentified kinase. The PLCγ1 dependent production of IP3 leads to increased calcium influx, Calcineurin activity, NFAT dephosphorylation and ultimately increased expression of NFAT-dependent genes, including RCAN1.1/1.4 and angiogenesis. CsA: Cyclosorin A, U73122: PLCγ1 inhibitor.

The ability of K15 to induce the phosphorylation of PLCγ1 and to induce angiogenic tube formation is relevant in virus-infected cells, since silencing of K15 by siRNA in HUVEC infected with recombinant KSHV, KSHV.219, inhibited phosphorylation of PLCγ1, NFAT1 dephosphorylation, RCAN1.1/1.4 expression and angiogenic tube formation following activation of the lytic cycle (figure 10). Furthermore, KSHVΔK15-infected HUVECs showed a significantly reduced angiogenic tube formation compared to KSHVwt-infected cells (figure 10 D–F). We therefore conclude that, in addition to previously reported viral mediators of angiogenesis such as K1 and vGPCR, the membrane protein K15 contributes to KSHV-induced angiogenesis. We acknowledge the limitations of this tissue culture based angiogenesis assay, which is however, widely used to study the effects of VEGF on angiogenesis. Recruitment and activation of PLCγ1 by K15 may represent an upstream signaling event that not only feeds into IP3/Ca^2+^ influx-mediated activation of Calcineurin and subsequent NFAT activation (figure 11), but could also be responsible, via DAG (figure 11), for the previously reported activation of the MEK/ERK and JNK pathways by K15 [50], [52].

Although we can detect K15 expression at the mRNA (figure 1) and protein (figure 10) level in KSHV-infected endothelial cells prior to the induction of the lytic cycle, we only observed moderate, or no, angiogenic tube formation in HUVECs that were infected with, respectively, BAC36-derived KSHVwt (figure 10 D, E) or rKSHV.219 (figure 10 A, B) prior to the activation of the lytic cycle. In contrast, we observed significant angiogenic tube formation upon activation of the lytic cycle along with increased K15 expression (figure 10). We therefore assessed the impact of K15, using siRNA and a KSHV K15 deletion mutant, in infected cells following activation of the lytic cycle in order to obtain more robust measurements. Others [18], [66] have reported the formation of angiogenic tubes in latently infected primary or immortalized endothelial cells. We believe that these differences are due to slight variations in the experimental protocols used: in our experiments, HUVECs were infected with rKSHV.219, BAC36-derived KSHVwt or KSHVΔK15 for 3–5 days before measuring angiogenic tube formation, since, in our experience, a significant number of ‘lytic’ cells (as assessed by RFP expression in the case of the rKSHV.219 virus [57]) is seen during the first 1–2 days after infection. We note that in the report by Sharma-Walia et al. [66], angiogenic tube formation was assessed 24 hours after infection. We attribute the fact that we observed moderate angiogenic tube formation in HUVECs infected with BAC36-derived KSHVwt prior to the induction of the lytic cycle, but did not in cells infected with rKSHV.219, to differences in the virus preparations used: owing to the low yield of BAC36 producer cell lines, these virus preparations have to be concentrated (ultracentrifugation) to a higher degree and may have resulted in a higher proportion of lytically infected cells three days after infection.

This raises the question whether K15 exerts its impact on angiogenesis in KS lesions in latently infected cells, cells that show a restricted pattern of lytic gene expression, or cells undergoing the full productive replication cycle. The K15 locus in the KSHV genome appears to be occupied by ‘active’ chromatin characterized by specific histone acetylation and methylation patterns (H3K9/K14ac, H3K4me3) [67] and K15 was originally described as expressed during latency, but upregulated during lytic replication [48]. This suggests that ‘basal’ expression of K15 in infected cells that do not undergo full lytic replication may be possible and that it is this type of cells with a ‘restricted’ lytic program that contributes to KSHV-mediated angiogenesis. K15 may therefore exert its angiogenic effect in the early stages of KS (when aberrant angiogenesis rather than spindle cells dominate the histology and limited lytic gene expression may play a role) or in a subpopulation of cells showing a restricted pattern of lytic gene expression. Answering this question would require the development of new reagents, in particular of monoclonal antibodies capable of detecting K15 protein in paraffin-embedded histology sections with high sensitivity and specificity.

Our observation that K15 ‘usurps’ the PLCγ1-Calcineurin-NFAT pathway and renders K15-expressing cells refractory to additional stimulation by VEGF (figure 6C) does not argue against an effect of VEGF, induced by other viral proteins, on KSHV-infected or neighbouring cells that do not express K15. Our results therefore do not exclude other, paracrine effects mediated by KSHV and K15, e.g. via K15-induced IL8 secretion [52], [58].

In summary our findings suggest that recruitment and activation of PLCγ1 to the SH2 and SH3- binding sites of K15 may represent an important facet in KSHV-induced angiogenesis and a putative target for the development of inhibitors that do not interfere with the physiological activation of PLCγ1 and that would offer the opportunity to inhibit the angiogenic effects of this virus.
